# Metaheuristic optimization of deep CNNs for multi-class diagnosis of cervical cancer and lymphoma

**DOI:** 10.1038/s41598-026-51619-3

**Published:** 2026-05-14

**Authors:** Ehab H. Abdelhay, Khaled Mohammed Elgamily, Walaa Omar El-Farouk Badr

**Affiliations:** 1 Department of Electronics and Communications Engineering, Faculty of Engineering, Mansoura University, Mansoura, 35516 Egypt; 2 Faculty of Engineering, Mansoura National University, Gamasa, 7731168 Egypt; 3 Department of Communications and Electronics Engineering, Mansoura Higher Institute of Engineering and Technology, Mansoura, 35516 Egypt; 4 Faculty of Artificial Intelligence and Information, Horus University-Egypt, New Damietta, 34517 Egypt

**Keywords:** Cervical cancer, Lymphoma, VGG-16, Optimization, Deep learning, Cancer, Computational biology and bioinformatics, Mathematics and computing

## Abstract

Accurate and early diagnosis of cancer is critical for determining effective treatment strategies and improving patient survival rates. However, automated multi-class cancer detection remains an enormous clinical and computational challenge due to the high visual heterogeneity within specific cancer classes and the morphological similarities across different types of malignancies. Deep convolutional neural networks (CNNs), particularly the VGG-16 architecture, offer robust feature extraction capabilities for medical imaging; yet, their diagnostic performance is heavily restricted by suboptimal hyperparameter tuning and inefficient feature utilization. To solve this problem, this article proposes a comprehensive, dual-strategy deep learning framework that integrates both pre-trained and fine-tuned VGG-16 models with six nature-inspired metaheuristic optimization algorithms. By employing the Whale Optimization Algorithm (WOA), Grey Wolf Optimizer (GWO), Particle Swarm Optimization (PSO), Genetic Algorithm (GA), Ant Colony Optimization (ACO), and Modified PSO (MPSO), the framework autonomously optimizes critical hyperparameters to maximize classification accuracy. The proposed methodology was rigorously evaluated on two complex imaging datasets: a five-class dataset for cervical cancer (a leading global cause of female cancer-related mortality) and a three-class dataset for lymphoma (a complex malignancy of the lymphatic system). The experimental results demonstrated that integrating pre-trained VGG-16 networks with metaheuristic optimizers significantly outperformed baseline models across both datasets. Notably, the Whale Optimization Algorithm (WOA) exhibited superior performance, achieving up to 100% in accuracy, precision, recall, and specificity during the testing phase for both datasets. These findings confirm that optimizing deep CNNs with metaheuristic algorithms provides a highly adaptable, reliable, and precise framework capable of resolving the complexities of high-dimensional multi-class cancer diagnosis.

## Introduction

Accurate cancer diagnosis is critical for selecting effective treatments and improving patient survival. Cervical cancer continues to be one of the most prevalent malignancies impacting women globally, with early detection being essential for enhancing prognosis and decreasing mortality rates^1^. In contrast, lymphoma encompasses a heterogeneous group of malignancies arising from the lymphatic system, distinguished by diverse morphological features and clinical presentations that pose challenges to accurate diagnosis^2^. The diverse visual patterns in medical imaging make reliable classification difficult. Variations within the same cancer type and similarities among different types of cancer frequently complicate automated analysis, thereby increasing the probability of misclassification. These challenges highlight the critical need for intelligent and dependable CAD (computer-aided diagnosis) systems capable of assisting healthcare professionals in making accurate and consistent decisions^3^. In recent years, deep learning methodologies, particularly convolutional neural networks (CNNs), have demonstrated exceptional effectiveness in analyzing medical data, including the detection and classification of diseases. The VGG-16 architecture is distinguished among the most commonly utilized models due to its deep hierarchical structure and its high efficacy in detecting salient visual features^4^. The field continues to evolve rapidly, with very recent work in 2025 introducing sophisticated hybrid optimization techniques. These include fast-flying particle swarm optimization, quantized orthogonal experimentation, and adaptive dimensional search-based algorithms designed to solve complex feature selection and neural network training problems^53–57^. Since acquiring large, annotated medical datasets is difficult, transfer learning has become an essential strategy for overcoming data scarcity. This framework allows networks to utilize generalized feature representations acquired from large, non-domain-specific datasets. The fine-tuning process, which utilizes pre-trained weights as a starting point and adapts them through additional training on a specific cancer dataset, enables the optimization of parameters to more effectively capture domain-specific features. This methodological approach has been shown to improve feature discriminability and model generalization relative to strategies that utilize frozen pre-trained layers independently^5^. Nonetheless, despite the recognized benefits of pre-trained and fine-tuned models, their classification efficacy may still be inadequate when confronted with the intrinsic complexity of multi-class oncology diagnostics. The performance of deep CNNs depends on the features extraction and the hyperparameters selection. This has led to the development of better CNN architectures, in which pretrained and fine-tuned models are changed and improved so that they can find task-specific patterns better while still being able to generalize. Metaheuristic algorithms enhance deep learning models by efficiently navigating complex search spaces^6^. Algorithms including the Whale Optimization Algorithm (WOA), Grey Wolf Optimizer (GWO), Particle Swarm Optimization (PSO), Genetic Algorithm (GA), and Ant Colony Optimization (ACO) which are based on natural and biological processes have shown a lot of promises for improving the optimization of deep learning systems. These methods are particularly beneficial for deep learning applications, as they facilitate the optimization of features, hyperparameters, and classification strategies independently of gradient-based techniques. Comprehensive comparative analyses assessing various optimizers across multiple datasets are notably limited, especially in the context of multi-class cancer diagnosis. The primary motivation for this work stems from the critical need to overcome the bottleneck of sub-optimal hyperparameter tuning in deep learning models applied to oncology. While pre-trained CNNs like VGG-16 have advanced medical image analysis, their diagnostic efficacy in distinguishing highly heterogeneous, multi-class cancers such as cervical cancer and lymphoma remains heavily restricted by manual or inefficient hyperparameter selection. Furthermore, there is a notable gap in the current literature regarding comprehensive, comparative evaluations of how different nature-inspired metaheuristic algorithms perform when optimizing these networks. Therefore, this study is motivated by the necessity to systematically identify the most robust, highly adaptable, and computationally efficient optimization strategy to maximize CNN performance, thereby providing a more reliable Computer-Aided Diagnosis (CAD) tool for clinical settings. Despite the success of deep learning in medical imaging, most studies still rely on default hyperparameter settings or only test a single optimizer on simple binary tasks. Because of this, a major research gap exists, as there is a lack of direct side-by-side comparisons of different metaheuristic algorithms for complex, multi-class cancer diagnosis. Our work directly addresses this gap by testing six nature-inspired algorithms head-to-head. By evaluating how well they optimize the VGG-16 network for multi-class cervical cancer and lymphoma datasets, we provide clear, evidence-based guidance on which optimization strategy yields the highest diagnostic accuracy. This article presents a comprehensive comparative framework that integrates six metaheuristic optimization algorithms WOA, GWO, PSO, GA, ACO, and a modified PSO (MPSO) with both pretrained and fine-tuned VGG-16 models to address these limitations. The proposed methodology is evaluated using two publicly available cancer datasets that have different levels of classification complexity: a five-class cervical cancer dataset and a three-class lymphoma dataset. This study provides a detailed evaluation of the impact of each optimization method on cancer classification performance by implementing various evaluation metrics. The proposed system consists of three main steps: (i) preprocessing the data, (ii) training and optimizing the VGG16 model, and (iii) Classify the different types of cancer. To address the limitations of current methodologies, the main contributions of this study are explicitly defined as follows:

1. A Comprehensive Multi-Algorithm Framework: Most current studies either rely on manual hyperparameter tuning or test a single optimizer in isolation. In contrast, the article suggested a framework that directly compares six different nature-inspired algorithms side-by-side to autonomously optimize the VGG-16 network, significantly reducing manual intervention in hyperparameter tuning.
2. Focusing on Complex, Multi-Class Datasets: Rather than evaluating the proposed model on basic binary classification tasks like many existing methods, the research explicitly tackles the challenging visual similarities found in complex multi-class clinical data, specifically targeting cervical cancer and lymphoma.
3. Establishing a Superior Performance Benchmark: the research empirically proves which optimization strategy works best for these specific medical challenges. By demonstrating that the Whale Optimization Algorithm (WOA) achieves a flawless 100% across all primary metrics, we provide a definitive benchmark that heavily outperforms standard baseline models.

The rest of this article is organized as follows: The literature review is summarized in section “Related work”. Section  “Materials and methods” provides a detailed analysis of the applied approaches and algorithms. The experimental results of the proposed method are presented in section “ Experimental results”. Finally, the research is concluded in section “Conclusion”.

## Related work

### Cancer classification using CNN

S. Lalitha et al.^7^ developed a framework for the detection and classification of cervical cancer using an optimized deep learning approach. The system employs modern metaheuristic algorithms to adapt ResNet hyperparameters for Pap smear analysis. The model improved classification performance by enhancing feature extraction without relying on manual cellular segmentation, demonstrating the effectiveness of automated tuning in overcoming data scarcity.

A. S. aly et al.^8^ proposed a hybrid optimized technique based on Dense Neural Networks for diagnosing malignant lymphoma. The methodology applied advanced algorithms, such as Harris Hawks Optimization, to optimize DenseNet architectures. Their approach significantly improved the classification of malignant subtypes, proving the validity of nature-inspired algorithms in complex hematological cancers.

D. Arifianto et al.^9^ developed a computerized diagnostic system utilizing a convolutional neural network for the classification of cervical cancer. A parameterized SqueezeNet model employing transfer learning has been optimized on a three-class dataset comprising 799 images. The model was trained with Caffe model and data augmentation, and it achieved an accuracy rate for classification of 98.41% which is better than MobileNet and SENet, and it also cut down on the cost and time needed for training, which shows that it is suitable for medical imaging applications which lack a lot of resources.

H. Alquran et al.^10^ proposed an automated framework for cervical cancer detection employing a novel deep learning architecture integrated with feature fusion from pre-trained networks. The system employs a multi-stage algorithm and combines deep features with SVM classifier, with an accuracy of 99.1% across 5 classes. By carefully selecting and integrating the most important features, it exceeds existing machine learning and deep learning methods, demonstrating high reliability on a substantial single-cell dataset.

M. Wu et al.^11^ presented an efficient CNN-based approach for classifying cervical cancer subtypes from H&E-stained cytological images, with the aim of supporting pathologists in resource-limited regions. By employing image augmentation techniques, the training dataset was substantially expanded, leading to a 3.85% improvement in classification performance and a maximum accuracy of 93.33%.

A. Ghoneim et al.^12^ illustrated a cervical cancer detection and classification framework based on convolutional neural networks, including comprehensive feature extraction with a machine learning classifier. Utilizing transfer learning and fine-tuning on the Herlev dataset, the proposed CNN–ELM method attained remarkably high accuracy in both binary and multi-class classifications, surpassing MLP- and AE-based alternatives. The findings indicate that combining CNN features with lightweight machine learning classifiers can yield precise and efficient diagnoses of cervical cancer, especially for early detection and clinical decision-making support.

A. Cibi et al.^13^ investigated a customized Convolutional Neural Network (CNN) integrated with a Capsule Network to autonomously categorize stages of cervical cancer from MR images. The model was designed with residual connections and optimized training settings to more effectively capture complex image features. It was trained on a large public dataset. The proposed method had an accuracy rate of 90.28% across eight stages of cancer.

M. Subramanian et al.^14^ presented an AI system for classifying eight types of cancer from histopathological images into multiple classes. The approach utilizes transfer learning with pre-trained CNNs such as MobileNet and DenseNet that are developed with Bayesian techniques. To prevent catastrophic forgetting while learning, the authors used the Learning without Forgetting (LwF) technique. This approach surpassed a great performance on the new oncology tasks and demonstrated a robust and versatile diagnostic tool.

S. L. Tan et al.^15^ developed an automated system for cervical cancer detection that employs pre-trained deep convolutional neural networks (CNNs) to tackle data scarcity and eliminate reliance on manual segmentation. The study evaluated 13 models using the Herlev Pap smear dataset and found that DenseNet-201 was the best architecture, achieving an accuracy of 87.02% in a seven-class classification task without the need for custom feature extraction. The study demonstrates that transfer learning can attain high accuracy with minimal training time, providing a practical and efficient foundation for automated detection in areas with limited resources.

W. Rahman et al.^16^ combined deep feature extraction with machine learning to classify leukemia subtypes. They have utilized pre-trained convolutional neural network architectures, such as ResNet50, to extract features. Then, they have reduced the number of dimensions and classified the data through principal component analysis (PCA) and a support vector classifier (SVC). They also applied Particle Swarm Optimization (PSO) and Cat Swarm Optimization (CSO) to enhance the classification. The proposed model had a classification accuracy of 99.84%, which means that it greatly improved the accuracy of diagnoses.

B. Sheng et al.^17^ introduced an automated system for lymphoma detection utilizing the Faster R-CNN object detection framework. To overcome the constraints of conventional pathological diagnosis, the authors initially developed a novel, high-quality dataset of blood cell images annotated with lymphoma cells, blasts, and lymphocytes. Through the fine-tuning of a pre-trained model on this dataset, their approach attained a lymphoma cell detection rate surpassing 96%, with a false detection rate under 13%, illustrating a notable enhancement in accuracy and efficiency for automated hematological cancer screening.

M. Ahmad et al.^18^ proposed a CNN-based approach for the automatic detection of metastatic cancer in histopathological lymph node images to assist pathologists with challenging diagnoses. Their model executed automated feature extraction and attained a remarkable average classification accuracy of 0.98 through image pre-processing and data augmentation on the PatchCamelyon dataset. This performance surpasses that of renowned architectures such as VGG-16 and ResNet-50.

J. Carreras et al.^19^ introduced a deep learning model for the complex histopathological differentiation between follicular lymphoma and reactive lymphoid hyperplasia in H&E-stained lymph node biopsies. The CNN based on ResNet architecture and trained with more than 1.4 million image patches, attained a patch-level accuracy of 99.80%. The research improved diagnostic transparency by utilizing explainable AI (XAI) methods, such as Grad-CAM and LIME, and confirmed robustness via patient-level partitioning.

M. Hamdi et al.^20^ developed a hybrid AI framework for diagnosing malignant lymphoma using whole-slide images. They utilized a multi-stage approach to enhance the accuracy of the diagnosis. The process started with improving the image using Gaussian and Laplacian filters, and then they employed the Gradient Vector Flow (GVF) algorithm to figure the cells out. Furthermore, they have used three main methods to extract features: pre-trained deep learning networks (MobileNet, VGG16, and AlexNet), combining features from hybrid DL architectures, and combining these deep features with color, shape, and texture descriptors. The framework then used the XGBoost and Decision Tree algorithms to optimize and sort these feature sets. Finally, they have applied the Ant Colony Optimization (ACO) algorithm to optimize the results. Their model MobileNet-VGG16 had reached an accuracy of 99.8% and an AUC of 99.43%.

S. Rajadurai et al.^21^ proposed a transfer learning framework for the multi-class classification of malignant lymphoma subtypes, specifically CLL, FL, and MCL. initially, the article examined VGG16, VGG19, DenseNet201, InceptionV3, and Xception as non-ensemble models. Then, to enhance performance, they utilized a stack-based ensemble model that combined InceptionV3 and Xception, achieving an outstanding diagnostic accuracy of 99%. Table 1 summarizes the most recent research articles that discuss the detection of cancer in medical images.

**Table 1:** Complete summary of the literature review of recent research articles in state of art of cancer detection in medical images.

Authors	Algorithm/Model	Strengths	Challenges / Limitations
S. Lalitha et al.^7^	ResNet + Metaheuristic Optimization	Enhanced feature extraction without requiring manual cellular segmentation	Evaluates only a single optimization algorithm on a specific task
A. S. aly et al.^8^	ResNet-50 + Whale Optimization Algorithm (WOA)	Automated hyperparameter tuning; proved highly effective for oncological prediction	Focused on one optimizer, leaving its comparative multi- class generalization unverified
D. Arifianto et al.^9^	Parameterized SqueezeNet (Transfer Learning) with data augmentation	Achieved 98.41% accuracy; computationally efficient, reducing training cost and time; suitable for resource- constrained environments	Relied on a relatively small dataset (799 images) compared to larger clinical repositories
H. Alquran et al.^10^	Hybrid Deep Learning with Feature Fusion & SVM Classifier	High reliability with 99.1% accuracy across 5 classes; integrates deep features for robust classification	Framework complexity increases with multi-stage feature fusion; focused on single-cell dataset analysis
M. Wu et al.^11^	CNN-based approach with image augmentation	Addressed data scarcity via augmentation (+ 3.85% performance boost); aids pathologists in resource- limited regions	The maximum accuracy of 93.33% is lower than other state-of-the-art models in this review
A. Ghoneim et al.^12^	CNN for feature extraction coupled with ELM (Extreme Learning Machine)	Surpassed MLP and AE-based methods; highly efficient for both binary and multi-class classification on the Herlev dataset	Performance is heavily dependent on the quality of feature extraction before the ELM classification stage
A. Cibi et al.^13^	Custom CNN integrated with Capsule Network and residual connections	Autonomously categorizes 8 stages of cancer; effectively captures complex spatial features from MR images	Determining 8 specific stages is complex, resulting in a lower accuracy (90.28%) compared to binary detection
M. Subramanian et al.^14^	Transfer Learning (MobileNet/ DenseNet) with Bayesian techniques & LwF	Addressed “Catastrophic Forgetting” using LwF; robust and versatile across new oncology tasks	Incorporating Bayesian techniques and LwF increases the architectural complexity of the training process
S. L. Tan et al.^15^	Transfer Learning (DenseNet- 201 selected from 13 models)	Eliminates need for manual segmentation or custom feature extraction; efficient training time	Accuracy (87.02%) is relatively low for a 7-class task; heavily reliant on pre- trained weights
W. Rahman et al.^16^	Hybrid: ResNet50 + PCA + SVC + Swarm Optimization (PSO & CSO)	Extremely high accuracy (99.84%); successfully combines deep features with nature-inspired optimization	High computational complexity due to the hybrid pipeline (CNN + Dimensionality Reduction + Optimization + SVM)
B. Sheng et al.^17^	Faster R-CNN Object Detection Framework	High detection rate (> 96%) with low false positives (< 13%); created a high-quality annotated blood cell dataset	Object detection is computationally heavier than classification; false detection rate still leaves room for optimization
M. Ahmad et al.^18^	CNN-based approach with augmentation (on PatchCamelyon dataset)	Average classification accuracy of 0.98; outperforms standard architectures like VGG-16 and ResNet-50	Efficacy is tied to the specific preprocessing and augmentation strategies applied to the patch dataset
J. Carreras et al.^19^	ResNet-based CNN with Explainable AI (Grad-CAM, LIME)	High patch-level accuracy (99.80%); trained on massive dataset (1.4 M patches); XAI ensures transparency	Patch-level accuracy requires careful validation to ensure it translates correctly to patient-level diagnosis
M. Hamdi et al.^20^	Hybrid: GVF Segmentation + DL Features + Hand-crafted Features + ACO Optimization	Exceptional accuracy (99.8%) and AUC (99.43%); integrates color, shape, and texture with deep learning	Extremely complex processing pipeline (Segmentation -> Hybrid Extraction -> XGBoost -> Ant Colony Optimization)
S. Rajadurai et al.^21^	Stack-based Ensemble Learning (InceptionV3 + Xception)	Achieved 99% accuracy; ensemble approach effectively combines strengths of multiple architectures	Higher computational resource consumption required to run multiple large models simultaneously

### Optimization techniques

S. B. U. Meeran et al.^22^ presented an AI-powered diagnostic system utilizing the Whale Optimization Algorithm (WOA) to enhance the ResNet-50 architecture for cancer prediction. The WOA was utilized to automatically set critical hyperparameters, significantly maximizing classification accuracy. Their findings demonstrated that WOA is highly effective in oncological applications, achieving superior metrics compared to unoptimized baseline models.

J. K. Deshmukh et al.^23^ developed a hybrid CNN–ACO–LSTM framework to identify lung cancer in CT scans. they have Utilized a combination of CNN algorithm for spatial feature extraction, Ant Colony Optimization (ACO) for hyperparameter optimization, and an LSTM for evaluation, the framework was able to classify data with 97.8% accuracy. The research demonstrates that the integration of meta-heuristic optimization with deep learning enhances the evaluation of high-dimensional medical data.

K. Rajwar et al.^24^ The authors thoroughly investigated more than 540 metaheuristic architectures (MAs) in extensive detail. The research examines the actual innovation of numerous emerging models, highlighting that many depend on insignificant parameter adjustments rather than significant computational transformations. To address this, the researchers came up with a new taxonomy based on control parameters. This enabled it to become more straightforward to group and rate these strategies.

W. Winarno et al.^25^ optimized the Convolutional Neural Network (CNN) for brain tumor detection. They utilized the Whale Optimization Algorithm (WOA) to automatically set the hyperparameters. The researchers improved training the inclusion of callbacks such as early stopping. The CNN-WOA model produced from this research performed exceptionally, achieving 99.6% accuracy across various tumor types.

A. Raza et al.^26^ implemented the MOB-CFPSO framework to address the difficulties of identifying diverse medical images. The authors developed a robust transfer learning model by combining Mobile-Net with a constriction factor-based Particle Swarm Optimization (PSO), enabling it to manage various modalities, including colored and monochrome datasets. Experimental findings on brain tumor MRI and diabetic retinopathy datasets demonstrated the model’s enhanced stability and accuracy. The method achieved an astonishing 99.86% validation accuracy for MRI evaluation.

J. O. Agushaka et al.^27^ explored the significant impact of initialization in population-based metaheuristic approaches. The article reviews several initialization strategies. The study evaluated their effectiveness and proposed avenues for further research. A comparative analysis is conducted to assess the impact of population size, total iterations, and ten initialization techniques on the performance of the bat algorithm (BA), Grey Wolf Optimizer (GWO), and butterfly optimization algorithm (BOA).

M. O. Oloyede et al.^28^ examined the relative effectiveness of nine Metaheuristic Optimization Algorithms (MOAs), including GWO and WOA, for medical image enhancement (MIE). The researchers employed the Fitness Computation Rate (FCR) to establish equitable benchmarking across Medpix datasets and analyzed results from 1,000 Monte Carlo trials. Although GWO and WOA exhibited slight empirical advantages, statistical analysis indicated no significant differences in image quality or processing speed among the evaluated methods. Thus, the results indicate that under elevated FCR conditions, selecting a particular MOA provides minimal comparative benefits for enhancement tasks.

B. Akay et al.^29^ presented an extensive study of metaheuristic methods used in deep neural network (DNN) design. Various DNN structures, optimization issues, encoding approaches, evolutionary operators, techniques for validation, and datasets are all covered. The study addresses the benefits, drawbacks, and potential directions of metaheuristic techniques by classifying them according to the type of problem. Table 2. summarizes the most recent research articles that discuss optimization techniques in medical images.

**Table 2:** Complete summary of the literature review of recent research articles in state of art of optimization techniques.

Authors	Algorithm/Model	Strengths	Challenges / Limitations
S. B. U. Meeran^22^	ResNet-50 + Whale Optimization Algorithm (WOA)	Automated hyperparameter tuning; proved highly effective for oncological prediction	Focused on one optimizer, leaving its comparative multi- class generalization unverified
J. K. Deshmukh et al.^23^	Hybrid CNN–ACO–LSTM Framework	Achieved 97.8% accuracy for lung cancer detection; effectively evaluates high- dimensional medical data by integrating spatial extraction (CNN) with temporal/sequence evaluation (LSTM)	The hybrid nature of the architecture increases computational complexity and the number of parameters required for training compared to single models
K. Rajwar et al.^24^	Taxonomy and Critical Review of 540 + Metaheuristic Architectures (MAs)	developed a new taxonomy based on control parameters to simplify classification; critical analysis of actual vs. perceived innovation	Highlighted a major issue in the field where many “new” models rely on insignificant parameter adjustments rather than genuine computational transformation
W. Winarno et al.^25^	CNN optimized with Whale Optimization Algorithm (WOA).	Automated hyperparameter tuning; achieved exceptional accuracy (99.6%) on brain tumors; callbacks (early stopping) improved training stability	Metaheuristic optimization for hyperparameter tuning adds significant computational overhead to the training phase compared to standard methods
A. Raza et al.^26^	MOB-CFPSO (Mobile-Net + Constriction Factor PSO)	High robustness across diverse modalities (color and monochrome); achieved 99.86% accuracy on MRI; transfer learning ensures efficiency	While Mobile-Net is lightweight, integrating PSO for optimization adds complexity to the convergence process
J. O. Agushaka et al.^27^	Comparative Analysis of Initialization Strategies (BA, GWO, BOA)	Evaluates the critical impact of population size and iterations; proposes specific avenues for improving algorithm initialization	Demonstrates that the performance of population- based metaheuristics is highly sensitive to the initial randomness and population settings
M. O. Oloyede et al.^28^	Comparative Study of 9 MOAs using Fitness Computation Rate (FCR)	Established equitable benchmarking standards; statistical analysis revealed no significant difference in quality/speed among methods	Concluded that under high FCR levels, selecting a specific modern MOA yields minimal comparative benefits
B. Akay et al.^29^	Comprehensive Survey of Metaheuristics in DNN Design	Covers optimization, encoding, and validation; classifies techniques by problem type; addresses benefits and drawbacks of DNN design	Addresses the broad complexity and lack of standardization in validating evolutionary operators and encoding approaches across different datasets

The synthesis of findings from the literature review, summarized in Tables 1 and 2, highlights that while standard CNN models have significantly improved diagnostic capabilities, they often face difficulties with the rigorous demands of hyperparameter tuning and feature selection across diverse medical datasets. Although recent studies from 2024 to 2025 demonstrate that integrating metaheuristic algorithms effectively resolves these issues, most current literature focuses on applying a single optimization algorithm to an isolated diagnostic task. This underscores the urgent need for a unified framework that systematically compares various optimization strategies—such as the six algorithms evaluated in this study to determine the most effective approach for complex, multi-class cancer detection. Unlike prior studies that often rely on a single optimization algorithm or handcrafted feature sets which can limit a model’s adaptability to different medical textures our approach systematically evaluates six distinct metaheuristics. This allows us to identify which search logic (e.g., the spiral-path logic of WOA versus the social-influence logic of PSO) is best suited for the high-dimensional challenges of multi-class oncological imaging.

## Materials and methods

This study proposed different metaheuristic optimization algorithms with pre-trained and fine-tunning VGG16 to enhance the performance of classification in Multi-Class Diagnosis of Cervical and Lymphoma Cancer. The suggested framework offers an outstanding approach for improving detection precision without necessitating the development of new structured algorithms. To ensure the effectiveness and robustness of the methodology, extensive testing is conducted across various cancer image datasets. This section discusses the suggested framework’s typical procedures including the main three steps: (i) data preprocessing, (ii) metaheuristic optimization & VGG-16 Fine tuning, and (iii) Cancer Classification.

### Datasets

(I) **Datasets description**

This study evaluates the proposed framework using two primary oncological datasets: **Cervical Cancer (SIPaKMeD)**^30,32^ and **Lymphoma**^31,33^, both sourced from the “Multi Cancer Dataset” repository.

- **Cervical cancer dataset**: Originally consisting of 966 high-resolution images across five subclasses (Dyskeratotic, Koilocytotic, Metaplastic, Parabasal, and Superficial-Intermediate), the data was expanded via augmentation to 25,000 images to enhance model robustness^30^.
- **Lymphoma dataset**: This set initially comprised 966 images across three subclasses (Chronic Lymphocytic Leukemia, Follicular Lymphoma, and Mantle Cell Lymphoma). Following identical augmentation procedures, the dataset was expanded to 15,000 images^31^.

Tables 3 and 4 summarize the technical specifications and the final distribution of the training, validation, and testing sets used in our experiments.

**Table 3:** Details on the data set used for training and evaluating the proposed models.

Dataset	No. of Subclasses	Subclasses	No of images utilized	Image size	Images type	Application
Cervical cancer^30^	5	Dyskeratotic Koilocytotic Metaplastic Parabasal Superficial-Intermediate	10,000	512 × 512 × 3	JPG	Medical Image Classification
Lymphoma^31^	3	Chronic Lymphocytic Leukemia Follicular Lymphoma Mantle Cell Lymphoma	12,000	512 × 512 × 3	JPG	Medical Image Classification

**Table 4:** Distribution of dataset.

Dataset	Train set /each class	Validation set/each class	Test set/each class	Total
Cervical cancer	1400	400	200	10,000
Lymphoma	2800	800	400	12,000

- **Cervical cancer dataset**.

**Fig. 1:**
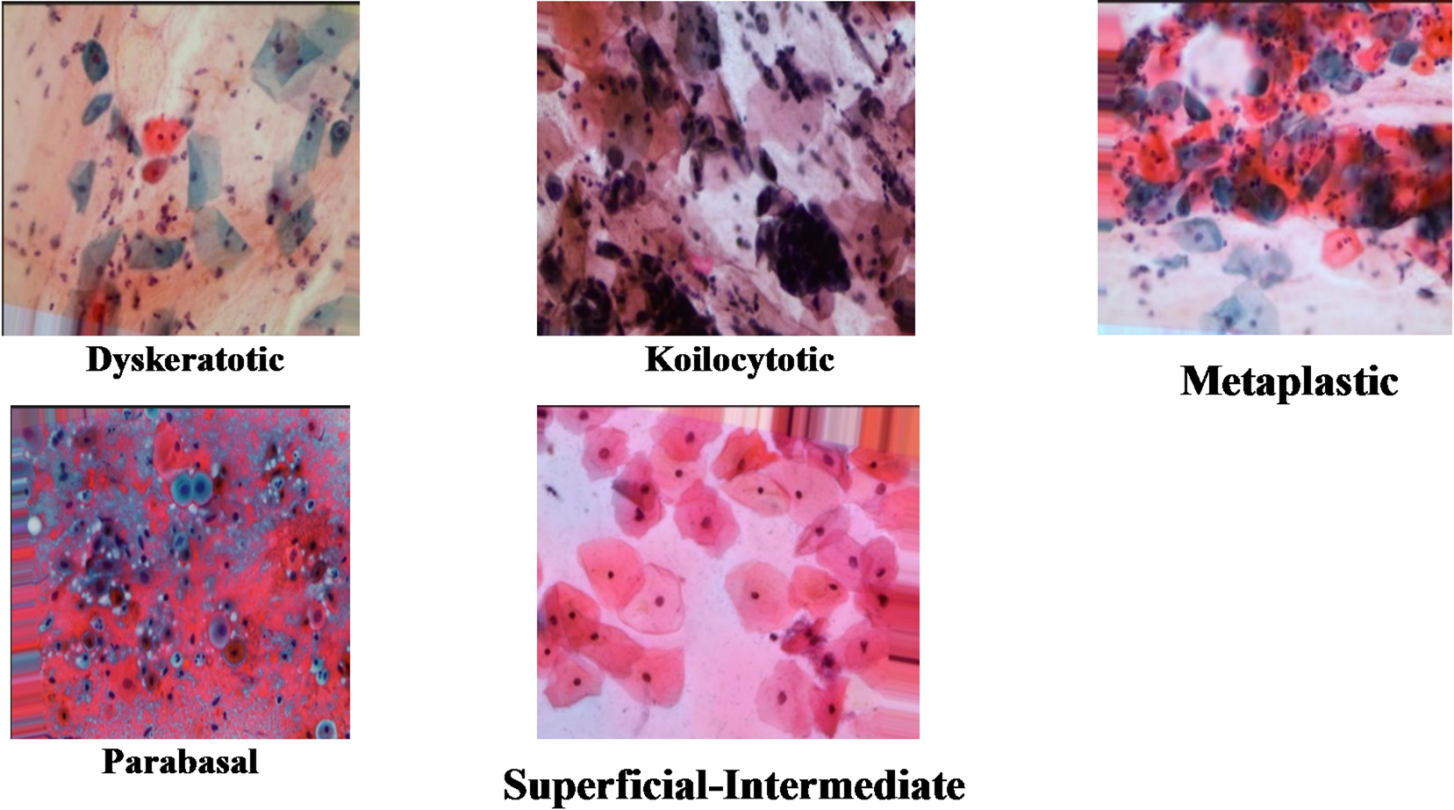
Samples of cervical cancer images.

Cervical cancer is the fourth most common cancer among women in the world. Detection of cervical cancer cells has played a very important role in clinical practice. The Cervical Cancer dataset Images obtained from Prahlad Mehandiratta dataset^32^. It consists of 966 images of 5 subclasses, 223 Dyskeratotic, 238 Koilocytotic ,271 Metaplastic, 108 Parabasal Superficial, and 126 Superficial-Intermediate. Then the images were augmented by using rotation up to 10-degree, width and height shift: Up to 10% of the total image size, shearing and zooming: 10% variation, brightness adjustment: ranges from 0.2 to 1.2 for varying light conditions, and randomly horizontal flip^30^. Samples of cervical cancer images are represented in Fig. 1.

(b) **Lymphoma dataset**

**Fig. 2:**
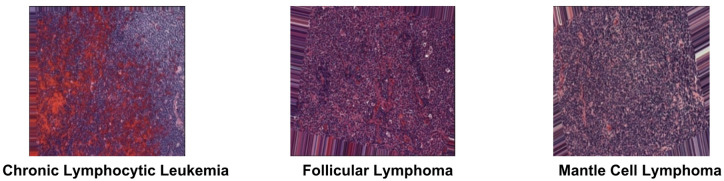
Samples of Lymphoma images.

Lymphoma consists of 966 images of 3 subclasses, 113 Chronic Lymphocytic Leukemia, 139 Follicular Lymphoma,122 Mantle Cell Lymphoma^33^. Then the images were augmented by rotation up to 10-degree, width & height shift: up to 10% of the total image size, shearing & zooming: 10% variation, brightness adjustment: ranges from 0.2 to 1.2 for varying light conditions, and randomly Horizontal Flip^31^. Samples of Lymphoma images are represented in Fig. 2.

(II) **Datasets preprocessing**

To prevent overfitting and improve generalization, we applied a standardized augmentation pipeline including 10° rotations, 10% width/height shifts, zooming, and brightness adjustments (0.2 to 1.2). Following augmentation, a two-step Preprocessing Pipeline was applied:

1. Spatial Resizing: All images were resized to a uniform 224 × 224 pixels to meet the architectural requirements of the VGG-16 model.
2. Normalization: Pixel intensities were rescaled from the standard [0, 255] range to [0, 1]. This step ensures numerical stability and accelerates gradient convergence during the optimization process.

### Methodology for classification

**Fig. 3:**
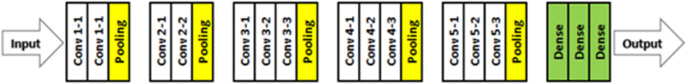
Structure of VGG-16 model.

This study employed transfer learning with both pre-trained and fine-tuned VGG-16 models to enhance performance and computational efficiency. Initially, the pre-trained model functioned as a feature extractor by the process of freezing its convolutional layers. Then, the original fully connected layers were substituted with custom dense layers made just for the target classification task. Furthermore, to ensure the model’s generalization and effective convergence, hyperparameter optimization was also employed to determine the best learning rate, optimizer type, batch size, and dropout rate. The article also systematically evaluated a number of optimizers, like Adam, RMSprop, and SGD, with different learning rate settings to ensure the stability of the training process. After the first step, the upper convolutional layers of the network were unfrozen and retrained with the best hyperparameters evaluated based on the metaheuristic optimizers on the pretrained VGG-16 to enhance the performance of the proposed model. The VGG-16 model was able to fit the target dataset well by using this combined method, which included feature extraction, hyperparameter optimization, and finally fine-tuning. All the mentioned techniques have ensured that the training phase is accurate, more generalized, and less costly. The Visual Geometry Group (VGG) at the University of Oxford made the VGG-16 architecture. It is a deep convolutional neural network architecture. It is well-known for being easy to operate, having a consistent design, and working well for many computer vision tasks. Figure 3 shows that the VGG-16 architecture has 16 trainable layers, 13 of which are convolutional layers and 3 of which are fully connected layers. The entire network uses small (3 × 3) convolution kernels and (2 × 2) max-pooling layers in the same structure on all layers. Because of this uniform structure^34^, VGG-16 can gradually pull out abstract and distinguishing features while keeping the architecture clear.

### Hyperparameter encoding and fitness function

To interface the metaheuristic algorithms with the VGG-16 architecture, each search agent (such as a whale in WOA or a particle in PSO) is assigned a position vector that represents a specific hyperparameter configuration. This configuration includes both continuous variables, such as the learning rate and dropout rate, and discrete variables, such as the batch size and the choice of optimizer (e.g., Adam, SGD, RMSprop). For discrete variables, the continuous output of the optimization algorithms is mapped using nearest-integer rounding to select the appropriate categorical value. To evaluate the quality of each search agent’s proposed configuration, a fitness evaluation mechanism is established. In this framework, the primary objective is to maximize the diagnostic capability of the VGG-16 model. Therefore, the fitness score assigned to each agent is the Validation Accuracy achieved after training the VGG-16 model for a defined number of epochs using that agent’s specific hyperparameter set. During each iteration, the metaheuristic optimizer records these fitness scores and updates the agents’ positions, systematically guiding the population toward the hyperparameter configuration that yields the highest validation accuracy without overfitting.

### Optimization algorithms

Metaheuristic optimization techniques, including the Whale Optimization Algorithm (WOA), Grey Wolf Optimizer (GWO), Particle Swarm Optimization (PSO), Genetic Algorithm (GA), Ant Colony Optimization (ACO), and Modified Particle Swarm Optimization (MPSO), are widely employed to optimize the hyperparameters selection of the pretrained and adaptively trained VGG-16 model. These optimization techniques were inspired by their proven effectiveness in addressing complex, high-dimensional problems in many fields including medical image analysis. PSO is recognized for its rapid convergence, rendering it highly suitable for swiftly identifying near-optimal solutions within extensive search spaces. GA’s genetic operators facilitate comprehensive search functionalities, enabling the exploration of an extensive array of solution spaces characterized by intricate cancer features. GWO sustains an equilibrium between exploration and exploitation, which is essential for preventing convergence to local optima during the training process. ACO mimics pheromone signaling to map out optimal trajectories within the feature space, while WOA is specifically favored for its bubble-net technique, which robustly handles high-dimensional complexity. The MPSO introduces structural tweaks designed to sustain diversity and prevent the search process from stagnating too early. These algorithms systematically explore the solution space to identify the optimal configuration for the deep learning models, enhancing classification accuracy while reducing computational redundancy. The proposed system employs metaheuristic optimization with VGG16 to the features of the cervical cancer and lymphoma datasets, thereby obviating the necessity for manual parameter tuning or algorithm adjustments. Within the realm of medical imaging and computer-aided diagnosis, the utilization of optimization algorithms serves as an effective approach for refining the parameters intrinsic to deep Convolutional Neural Networks (CNNs). Researchers aim to enhance the performance of the VGG-16 model by autonomously tuning essential hyperparameters to address the heterogeneity within cancer classes and the similarities across different classes. Table 5 summarizing the comparison of the metaheuristic optimization algorithms used in the proposed framework.

**Table 5:** The comparison of the proposed metaheuristic optimization algorithms.

Optimizer	Optimization process	Key Strength
(WOA)	Simulates the way humpback whales hunt in bubble nets	Great performance in high-dimensional spaces and complex feature maps
(GWO)	Mimics the grey wolf leadership hierarchy and hunting mechanism	Accurate exploration and exploitation to avoid local optima
(PSO)	Based on the social behavior of bird flocking or fish schooling	Fast convergence; ideal for quickly locating near-optimal regions in large search spaces
(GA)	applies evolutionary operators (mutation, crossover, selection)	Extensive search capabilities; allows for the exploration of a wide diversity of potential solutions
(ACO)	Uses pheromone trails to mimic how ants search for food	Effective pathfinding and discrete optimization; useful for navigating feature dependencies
(MPSO)	An updated version of PSO with adaptive adjustments	Improved population diversity to prevent premature convergence common in standard PSO

(I) **Whale Optimization Algorithm (WOA)**

The Whale Optimization Algorithm (WOA) emulates the characteristic social conduct and foraging methodologies of humpback whales, particularly their “bubble-net” feeding tactic. This approach simulates the hunting behavior of humpback whales, specifically their technique of encircling prey and generating spiral bubble nets to capture them^35^. The algorithm initiates by designating the current leading candidate solution as the target prey, predicated on the assumption that it represents the optimal position in proximity to the true optimum^36^. Following search agents, depicted here as whales, strive to modify their positions in relation to the most efficient search agent. The encompassing behavior is mathematically represented by the following Eqs. (1–5)^35,36^:

1 $$D=\left| {~C~.~{X^*}\left( t \right) - X\left( t \right)} \right|$$

2 $$X\left( {t+1} \right)={X^*}\left( t \right) - A.D~~~~~~~$$

Where t is the iteration number, ${X^*}$ describes the best solution position vector obtained, *X* the search agent position agent, finally A and C are the coefficient vectors and calculated as Eqs. (4–5).

3 $$A=2a~.~r - a$$

4 $$C=2~.~r$$

Where a descends from 2 to 0 across the iterations to encourage both exploration and exploitation. r is a random vector in the range [0, 1]. The algorithm uses a spiral equation to demonstrate the helix-shaped path of the whales as they get closer to their prey, such as in the bubble-net attack. This behavior is represented by the following Eq. (5).

5 $$X\left( {t+1} \right)=D^{\prime}.~{e^{bl}}.\cos \left( {2\pi l} \right)+{X^*}\left( t \right)$$

Where $D^{\prime}=~\left| {~{X^*}\left( t \right) - X\left( t \right)} \right|$ denotes the distance between the whale and the prey, *b* characterizes the shape of the logarithmic spiral, and *l* is a random variable within the interval [-1, 1]. The algorithm presumes a 50% likelihood of alternating between the shrinking encircling mechanism and the spiral model to update the whales’ positions.

(II) **Grey Wolf Optimizer (GWO)**

The Grey Wolf Optimizer (GWO) is a metaheuristic optimization algorithm which employs the social hierarchy and hunting strategies of grey wolves as its foundation. It utilizes the leadership hierarchy of wolves to systematically search through complex search spaces to find the best solutions. GWO is a promising way to improve the accuracy and efficiency of computer vision classification applications by optimizing their parameters and settings in the field of medical images classification. The GWO has three main steps: initialization, updating, and selection. In the initialization phase, a random set of solutions is made in the search space. Each solution stands for one wolf in the pack. A grey wolf’s mathematical location (or solution) can be described as ${x_i}=\left( {{x_{i1}},{x_{i2}},{\mathrm{~}}{x_{i3}}, \ldots ,{x_{iD}}} \right)$, where i = 1,2, ., N (N is the total number of wolves or population size) and D is the problem dimension^37^. Prey is the best solution the algorithm is seeking for where $\alpha$ is the Fitness Solution, $\beta$ is the second-best solution, $\delta$ is the third-best solution, and $\boldsymbol{\omega}$ is the rest of the solutions. The following formula, Eqs. (6–12)^38^, mathematically represent the social hierarchy of grey wolves. The initial step occurs when grey wolves surround their prey^38^.

6 $$\vec {D}=\left| {\vec {C} \cdot \overrightarrow {{P_t}} \left( i \right) - ~\vec {P}\left( i \right)} \right|$$

7 $$\vec {P}\left( {i+1} \right)=\overrightarrow {{P_t}} \left( i \right) - \vec {D} \cdot \vec {A}$$

Where, $\vec {A}$ and $\vec {C}$ are the coefficients vectors, ${P_t}$ is the position of the target prey, $\vec {P}$ is the gray wolf position vector, and *i* is the iteration number. $\vec {C}$ and $\vec {A}$ can mathematically described as^38^:

8 $$\vec {A}=2\vec {a} \cdot \overrightarrow {{r_1}} - ~\vec {a}{\mathrm{~}},{\mathrm{~}}\vec {a}=2\left( {\frac{{1 - i}}{I}} \right)$$

9 $$\vec {C}=2 \cdot \overrightarrow {{r_2}}$$

Where $\vec {a}$ Linearly reduced over the iterations between 2 and zero, *i* is the number of the iteration, I is the iterations maximum number and $\overrightarrow {{r_1}}$and $\overrightarrow {{r_2}}$ are random vectors within the range of [0–1].

10 $$\:\overrightarrow{{D}_{\alpha\:}}=\:\left|\overrightarrow{{C}_{1}}\cdot\:\overrightarrow{{P}_{\alpha\:}}-\:\overrightarrow{P}\right|,\:\overrightarrow{{D}_{\beta\:}}=\:\left|\overrightarrow{{C}_{2}}\cdot\:\overrightarrow{{P}_{\beta\:}}-\:\overrightarrow{P}\right|,\:\overrightarrow{{D}_{\delta\:}}=\:\left|\overrightarrow{{C}_{3}}\cdot\:\overrightarrow{{P}_{\delta\:}}-\:\overrightarrow{P}\right|$$

11 $$\:\overrightarrow{{P}_{1}}=\overrightarrow{{P}_{\alpha\:}}-\overrightarrow{{A}_{1}}\cdot\:\overrightarrow{{D}_{\alpha\:}}\:,\:\overrightarrow{{P}_{2}}=\overrightarrow{{P}_{\beta\:}}-\overrightarrow{{A}_{2}}\cdot\:\overrightarrow{{D}_{\beta\:}},\:\overrightarrow{{P}_{3}}=\overrightarrow{{P}_{\delta\:}}-\overrightarrow{{A}_{3}}\cdot\:\overrightarrow{{D}_{\delta\:}}$$

12 $$\:\overrightarrow{P}\left(t+1\right)=\:\frac{\overrightarrow{{P}_{1}}+\overrightarrow{{P}_{2}}+\overrightarrow{{P}_{3}}}{3}$$

**Fig. 4:**
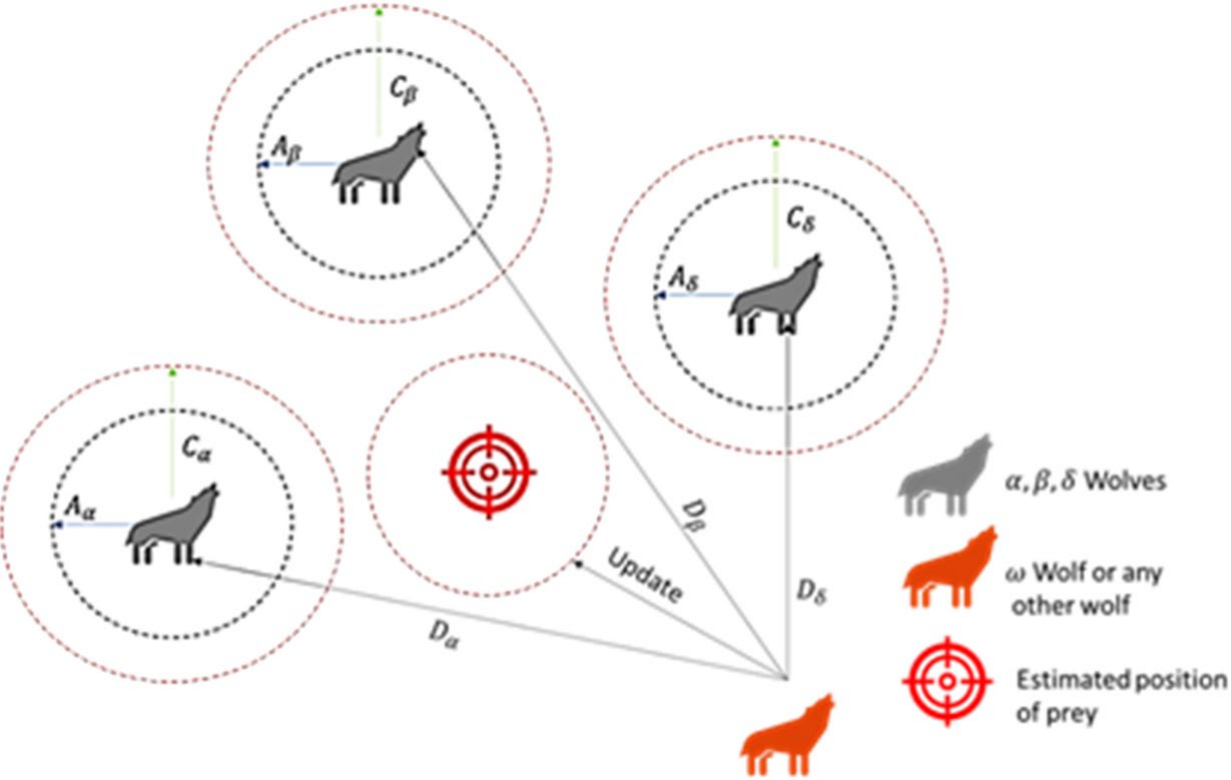
The Grey Wolf’s behavior of hunting.

Figure 4 represents the social hierarchy and hunting strategies of grey wolves.

(III) **Particle Swarm Optimization (PSO)**

Particle Swarm Optimization (PSO) is a popular method of metaheuristic optimization that is based on how groups of birds or fish move together. Particle Swarm Optimization (PSO) utilizes a group of possible solutions, called particles, each with its own position and speed, to search for space with more than one dimension. The basic idea is that these particles will gradually improve until they reach the best possible state, based on their own and each other’s experiences^39^. To make the mathematical model of Particle Swarm Optimization (PSO), employ Eq. (13) to modify the velocity and Eq. (14) to modify the position^40^.

13 $$\:{\vartheta\:}_{i,d}\left(t+1\right)=\:\omega\:\times\:{\vartheta\:}_{i,d}\left(t\right)+\:{c}_{1}\times\:{r}_{1}\times\:\left({{P}_{b}}_{i,d}-{P}_{i,d}\left(t\right)\right)+{c}_{2}\times\:{r}_{2}\times\:\left({{G}_{b}}_{d}-{P}_{i,d}\left(t\right)\right)$$

14 $$\:{P}_{i,d}\left(t+1\right)={P}_{i,d}\left(t\right)+\:{\vartheta\:}_{i,d}\left(t+1\right)\:$$

Where,, $\:{c}_{1}$ and $\:{c}_{2}$ are the acceleration constants, $\:\omega\:$ is the inertia weight for local and global exploration balancing, $\:{\vartheta\:}_{i,d}$ is the initial velocity, $\:{r}_{1}$ and $\:{r}_{2}$ are random numbers in the range (0,1), $\:{P}_{i,d}$ is the initial position for each i which is the particle and the dimension is d, $\:{P}_{b}$ is the agent’s best solution, and the global best solution is $\:{G}_{b}$.

(IV) **Genetic Algorithm (GA)**

Genetic Algorithm (GA) is an effective method for identifying the best solution. It is based on genetics and the idea of natural selection. GA mimics the processes of evolution to quickly search through complex solution spaces^41^. The algorithm starts with a set of possible solutions and then utilizes an objective function to determine their performance. The GA framework includes chromosome visualization, selection, crossover, mutation, and the calculation of fitness functions. The GA method starts by making a population of n chromosomes. The fitness of each chromosome is evaluated and based on the fitness score two chromosomes are chosen. To make children, these chosen chromosomes are crossed over at one point. This offspring then undergoes a uniform mutation process, resulting in the creation of new offspring. This new offspring is added to the population. The operations of selection, crossover, and mutation continue until the new population is completed. GA adapts its method of search based on the individuals it examines, which lets it find many optimal solutions. It maintains the diversity of the population by changing the original schema with a new version. The next three Eqs. (15–17) show the main parts of GA mathematically^42^.

15 $$\:{S}_{p}\left(x\right)=\frac{f\left(x\right)}{\sum\:_{i=1}^{N}f\left(x\right)}$$

Where $\:{S}_{p}$ is the selection probability, and $\:N$ is the solution size, $\:f\left(x\right)$ is the fitness function, and $\:x$ is the individual solution.

16 $$\:{x}_{offspring}=concat({x}_{\mathrm{1,1}:k},\:{x}_{2,k+1:N})$$

Where,, $\:k$ is the point of the cross-over and $\:N$ is the length of the solution.

17 $$\:{x}_{mutated}=\:\left\{\begin{array}{c}{x}_{offspring}\:\:\:\:\:\:\:\:\:\:\:\:\:\:\:\:\:\:\:\:\:\:\:\:\:\:with\:probaility\:1-{p}_{m}\\\:mutate\left({x}_{offspring}\right)\:\:\:\:\:\:\:\:\:\:\:\:\:\:\:with\:probaility\:{p}_{m}\end{array}\right.$$

Where, $\:{x}_{offspring}$ is the offspring, and $\:{p}_{m}$ parameter of probability which is set before running the genetic algorithm.

(V) **Ant Colony Optimization (ACO)**

The Ant Colony Optimization (ACO) algorithm is a probabilistic method based on the behavior which ant colonies follow in searching for food. For example, it is based on how ants can find the shortest path between their nest and a food source^43^. This method uses “pheromones,” chemical signals that ants release to guide other ants in the colony. A group of artificial ants moves through a graph that shows the states of the problem to find solutions in the computational model. Two primary factors affect the probability of an ant ($\:k$) moving from node (n) to node (m): the pheromone concentration on the edge $\:{\tau\:}_{nm}$ and a heuristic value $\:{\aleph\:}_{nm}$, that determines desirability the move (often defined as the inverse of the distance). The transition probability is represented as follows in Eq. (18)^43,44^.

18 $$\:{P}_{nm}^{k}=\frac{{\left({\tau\:}_{nm}\right)}^{\alpha\:}{\left({\aleph\:}_{nm}\right)}^{\beta\:}}{\sum\:_{l\in\:{N}_{n}^{k}}{\left({\tau\:}_{nl}\right)}^{\alpha\:}{\left({\aleph\:}_{nl}\right)}^{\beta\:}}\:,\:\mathrm{i}\mathrm{f}\:j\in\:\:{N}_{n}^{k}\:$$

Here, $\:{N}_{n}^{k}$ is the group of nodes that ant k can move to from node n. The parameters $\:\alpha\:$ and $\:\beta\:$ demonstrate the importance of the pheromone trail in comparison to the heuristic information. After all the ants have come up with their own solutions, the pheromone trails are updated to demonstrate the solutions accuracy. This update process has two phases: evaporation, which stops pheromones from building up excessively and deposition, in which ants leave new pheromones along the paths they have traveled. Equation (19) describes the global pheromone update rule^43^.

19 $$\:{\tau\:}_{nm}\left(t+1\right)=\left(1-\:\epsilon\:\:\right)\:\cdot\:\:{\tau\:}_{nm}\left(t\right)+\:\sum\:_{k=1}^{i}{\varDelta\:\tau\:}_{nm}^{k}\:$$

Where $\:\epsilon\:$ presents the rate of evaporation (where 0 < $\:\epsilon\:$ < 1), and $\:{\varDelta\:\tau\:}_{nm}^{k}$ presents the pheromone amount deposited by the kth ant, typically correlated to the efficiency of the solution it developed. This feedback mechanism guarantees that, over time, the colony progresses toward the most optimal route.

(VI) **Modified Particle Swarm Optimization (MPSO)**

Modified Particle Swarm Optimization (MPSO) improves upon the standard PSO framework to mitigate problems of premature convergence and stagnation at local optima, which are common challenges in high-dimensional optimization. Although conventional PSO utilizes static parameters, MPSO enhances search efficiency through the incorporation of an adaptive inertia $\:\omega\:$ omega and an acceleration factor $\:\alpha\:$. The adaptive inertia weight $\:\omega\:$ diminishes linearly throughout the execution process, transitioning the emphasis from global exploration in the initial phases to local exploitation in the subsequent stages. The acceleration factor actively pushes particles toward the best global position based on their own history^45^. This change is meant to make the search process easier and cut down on the time it takes to find the best hyperparameters for deep learning models. Equation (20) defines the update rule in MPSO^45^.

20 $$\:{\vartheta\:}_{i}^{t+1}=\:\omega\:\left(t\right)\cdot\:\:{\vartheta\:}_{i}^{t}+\:{c}_{1}\cdot\:{r}_{1}\cdot\:({P}_{best,i}-{x}_{i}^{t}{)+c}_{2}\cdot\:{r}_{2}\cdot\:\left({G}_{best}-{x}_{i}^{t}\right)-\:\alpha\:({G}_{best}-\:{P}_{best,i})$$

Where, $\:{c}_{1}$ and $\:{c}_{2}$ are the coefficients of acceleration, $\:{r}_{1}$ and $\:{r}_{2}$ are random vectors in range [0–1], $\:\alpha\:\:$is the acceleration factor, $\:{G}_{best}$ is the global best solution and $\:{P}_{best,i}$ is the local best solution. The updated velocity position $\:{x}_{i}^{t+1}$ for particle i is represented in equations (21).

21 $$\:{x}_{i}^{t+1}={x}_{i}^{t}+{\vartheta\:}_{i}^{t+1}$$

### Integration workflow of the dual-strategy framework

The integration of VGG-16 with the metaheuristic algorithms operates through a systematic, nested loop architecture. The operational workflow proceeds as follows:

**Step 1**: Initialization: The target dataset (Cervical or Lymphoma) is preprocessed, augmented, and divided into training, validation, and test subsets.

**Step 2**: Population Setup: A population of *N* = 5 search agents is initialized randomly within the predefined hyperparameter bounds.

**Step 3**: Pre-trained Optimization Phase: For a maximum of T = 3 iterations, each agent’s hyperparameter set is injected into a VGG-16 model (with frozen convolutional bases). The model is trained on the training set, and the validation accuracy is returned to the optimizer as the fitness score.

**Step 4**: Metaheuristic Update: The chosen optimization algorithm (e.g., WOA, GWO) updates the agents’ positions based on the highest recorded fitness score.

**Step 5**: Fine-tuning Phase: Once the global best hyperparameter configuration is identified, the upper convolutional layers of the VGG-16 are unfrozen. The model is then fully retrained (fine-tuned) using these optimal hyperparameters to adapt the feature extraction process specifically to the oncological dataset.

**Step 6**: Final Evaluation: The final optimized model is evaluated against the unseen test dataset to generate the ultimate accuracy, precision, recall, and specificity metrics.

### Computational complexity analysis

The overall computational complexity of the proposed framework is governed by two primary factors: the execution time of the metaheuristic algorithm and the evaluation time of the VGG-16 fitness function. Let N represent the population size (number of search agents), T denote the maximum number of iterations, and D represent the dimension of the search space (the number of hyperparameters being optimized, which is D in this study).

The computational time for initializing the population is O(N×D). During the optimization phase, updating the positions of the search agents across algorithms like WOA, GWO, and PSO requires O(T×N×D). However, evaluating the fitness of each agent requires training in the pre-trained VGG-16 model for a set number of epochs. If the computational cost of training CNN is denoted as O(C_CNN_), the total complexity of the proposed framework can be formulated as:

$${\mathrm{O}}\left( {{\mathrm{Total}}} \right)\,=\,{\mathrm{O}}({\mathrm{N}} \times {\mathrm{D}})\,+\,{\mathrm{O}}({\mathrm{T}} \times {\mathrm{N}} \times ({\mathrm{D}}\,+\,{{\mathrm{C}}_{{\mathrm{CNN}}}}))$$

Because the time required to train a deep neural network is vastly greater than the time required to update the metaheuristic positions (C^CNN^ > > D), the asymptotic computational complexity fundamentally simplifies to O(T×N×C_CNN_). By deliberately restricting the population size (*N* = 5) and iterations (T = 3) during the hyperparameter search phase, the proposed framework strategically minimizes the T×N multiplier. This ensures that the computational overhead remains highly manageable while successfully identifying the global optimum for the final fine-tuning phase.

## Experimental results

This section presents the outcomes of utilizing metaheuristic-based hyperparameter tuning to VGG-16. To extensively evaluate the effectiveness of the proposed framework, the study employed two separate medical imaging datasets: a cervical cancer dataset containing five diagnostic classes and a lymphoma dataset featuring three classification categories. A thorough experimental approach was implemented, comprising twelve distinct tests for each dataset to evaluate both training and validation accuracy. These twelve experiments were organized into two distinct methodological phases:

- Pre-trained Model Evaluation: Six experiments were performed utilizing the pretrained VGG-16 model combined with six metaheuristic algorithms (WOA, GWO, ACO, PSO, GA, and MPSO).
- Evaluation of the Fine-Tuned Model: The remaining six experiments were conducted utilizing the fine-tuned VGG-16 model. This phase utilized the optimal hyperparameters determined during the pretrained model assessments to further enhance performance.

A comprehensive search space was created for the hyperparameters, allowing each metaheuristic algorithm to explore a wide array of values and adaptively produce diverse parameter configurations. Through this iterative process, the algorithms attained optimal configurations that enhanced classification performance.

### Experimental setup and parameter configuration

This study utilized an efficient hyperparameter tuning strategy to enhance the VGG-16 model’s diagnostic accuracy. To ensure consistency across experiments, several core parameters remained fixed: the input image size was set to 224 × 224 pixels, and the training length was defined as 5 epochs for the cervical cancer dataset and 20 epochs for the lymphoma dataset.

**Hardware and software environment**: All computational procedures were conducted using Python 3.9 within the Google Colab environment, utilizing the high-performance computing capabilities of an NVIDIA A100 GPU to enable the training and validation of the deep learning models. The framework was implemented using the TensorFlow and Keras libraries for the VGG-16 architecture, with Scikit-learn used for data partitioning and metric calculation.

**Statistical validation and robustness**: To ensure the stability and reliability of the reported results, a robust statistical validation framework was employed. All experiments were conducted using five-fold cross-validation, where the dataset was partitioned into five subsets to ensure that every image was utilized for both training and testing. This process minimizes data selection bias and provides a more accurate representation of the model’s generalization capability. **Hyperparameter search space**: This study utilized an efficient hyperparameter tuning strategy to enhance the VGG-16 model prediction accuracy. To ensure reliability through experiments, specific hyperparameters remain unchanged during the training phase. The input image size was set to 224 × 224 pixels. Also, the length of the training was set based on the dataset: 5 epochs were used for the cervical cancer dataset and 20 epochs were utilized for the lymphoma dataset. Due to the significant computational overhead associated with these experiments, the optimization algorithm was constrained to a population size of 5 and limited to 3 iterations. Despite these constraints, a comprehensive search space was explored to identify the ideal configurations. The research concentrated on altering essential hyperparameters, such as the learning rate, dropout rate, batch size, and the optimization algorithm utilized. To ensure full reproducibility of our optimization results, the search space and specific parameter ranges for all six metaheuristic algorithms (WOA, GWO, PSO, MPSO, GA, and ACO) are detailed in Table 6. This includes the bounds for the learning rate, dropout rate, and batch size, as well as the specific internal coefficients used to govern the exploration and exploitation phases of each nature-inspired optimizer.

**Table 6:** The ranges of the optimized parameters.

Parameter	Values
Learning rate	$\:1\times\:{10}^{-2},\:1\times\:{10}^{-3},\:\:1\times\:{10}^{-4},\:\:1\times\:{10}^{-5}$
Optimizer type	*Adam*,* N Adam*,* SGD*,* RMSprop*
Dropout value	*0.1*,*0.3*,* 0.5*,* 0.7*
Batch sze	*16*,* 32*,* 64*,* 128*
Epochs	5 (Cervical cancer) / 20 (lymphoma)
Input image size	224 × 224

**Algorithm settings and justification**: to ensure the reproducibility of the optimization process, the internal parameters for each metaheuristic algorithm (WOA, GWO, PSO, GA, ACO, and MPSO) were set to their standard default configurations, as presented in Table 7. These settings, such as the linearly decreasing inertia weights in PSO and the convergence parameters in GWO, were chosen to maintain a proper balance between global exploration and local exploitation within the restricted search budget of 3 iterations. By utilizing these established defaults, we ensure that the performance gains are attributed to the algorithms’ inherent search logic rather than manual bias in parameter tuning, providing a stable and reliable benchmark for oncological image classification.

**Table 7:** Internal parameter settings for metaheuristic optimizers.

Algorithm	Internal parameter	Value / Logic	Justification
WOA	Spiral shape (b)	1.0	Defines the logarithmic spiral path of whales
	Convergence (a)	2 to 0 (Linear)	Balances exploration (2) and exploitation (0)
GWO	Control parameter (a)	2 to 0 (Linear)	Mimics the narrowing search space of a hunt
PSO	Inertia weight (w)	0.9 to 0.4	High w for exploration; low w for local refinement
	Accel. coeffs (c1, c2)	2.0, 2.0	Standard balance between personal and social influence
GA	Crossover rate (P_c_)	0.8	Ensures high recombination of successful traits
	Mutation rate (P_m_)	0.05	Prevents premature convergence to local optima
ACO	Pheromone/Heuristic (α, β)	1.0, 2.0	Standard weighting for path selection logic
	Evaporation rate (ρ)	0.5	Controls the “memory” of previous successful paths
MPSO	Velocity Clamping	Dynamic	Prevents “swarm explosion” in high-dimensional spaces

To ensure the robustness and reliability of the proposed framework, each of the twelve experiments was executed independently over three separate runs with random weight initializations. The performance metrics presented in this section, including Accuracy, Precision, Recall, and F1-Score, represent the mean values obtained across these independent runs. The standard deviation was also recorded to quantify the variance. For instance, the proposed WOA-VGG16 model exhibited a remarkably low standard deviation (0.002) across all runs, confirming its stability and the reproducibility of the classification outcomes.

### Performance metrics

The evaluation of operational effectiveness in the classification of medical images includes several essential metrics, including Accuracy, Precision, Recall, F1-score, and Specificity. These metrics collectively evaluate the model’s ability to categorize various images, offering detailed information about its performance. True positives (TP) refer to objects correctly identified as members of the target class, while true negatives (TN) denote objects accurately recognized as non-members. False positives (FP) are instances that are incorrectly identified as members of the target class, whereas false negatives (FN) are instances that belong to the target class but are erroneously classified as outside it. Consequently, a detailed overview of the evaluation metrics is provided in Table 8^46,47^.

**Table 8:** Evaluation metrics description.

Metric	Abbreviations & formula	Explanation
Accuracy (ACC)	$\:ACC=\:\frac{TP+TN}{TP+FN+TN+FP}$	Evaluate the total number of the successful classifications divided by the overall number of classifications done by the algorithm
Recall	$\:Recall=\:\frac{TP}{TP+FN}$	Evaluate the ratio of the true positively classified samples to the total number of positively classified samples
Specificity (SPEC)	$\:SPEC=\:\frac{TN}{TN+FP}$	Assess the system’s capacity for precise prediction of unfavorable outcomes
Positive predictive value (P- value)	$\:P-Value=\:\frac{TP}{TP+FP}$	The number of correctly classified true positives is compared to all positive samples
Negative predictive value (N-value)	$\:N-Value=\:\frac{TN}{TN+FN}$	The number of incorrectly classified true negative is compared to all negative samples
F1 - score	$\:F1-Score=\:2\times\:\frac{precision\:\times\:recall}{precision+recall}$	the precision and recall weighted mean
Precision	$\:Precision=\:\frac{TP}{TP+FP}$	It is calculated as the ratio of all the algorithm’s positive classifications divided by the total number of real positive classifications

### Experiments

**Fig. 5:**
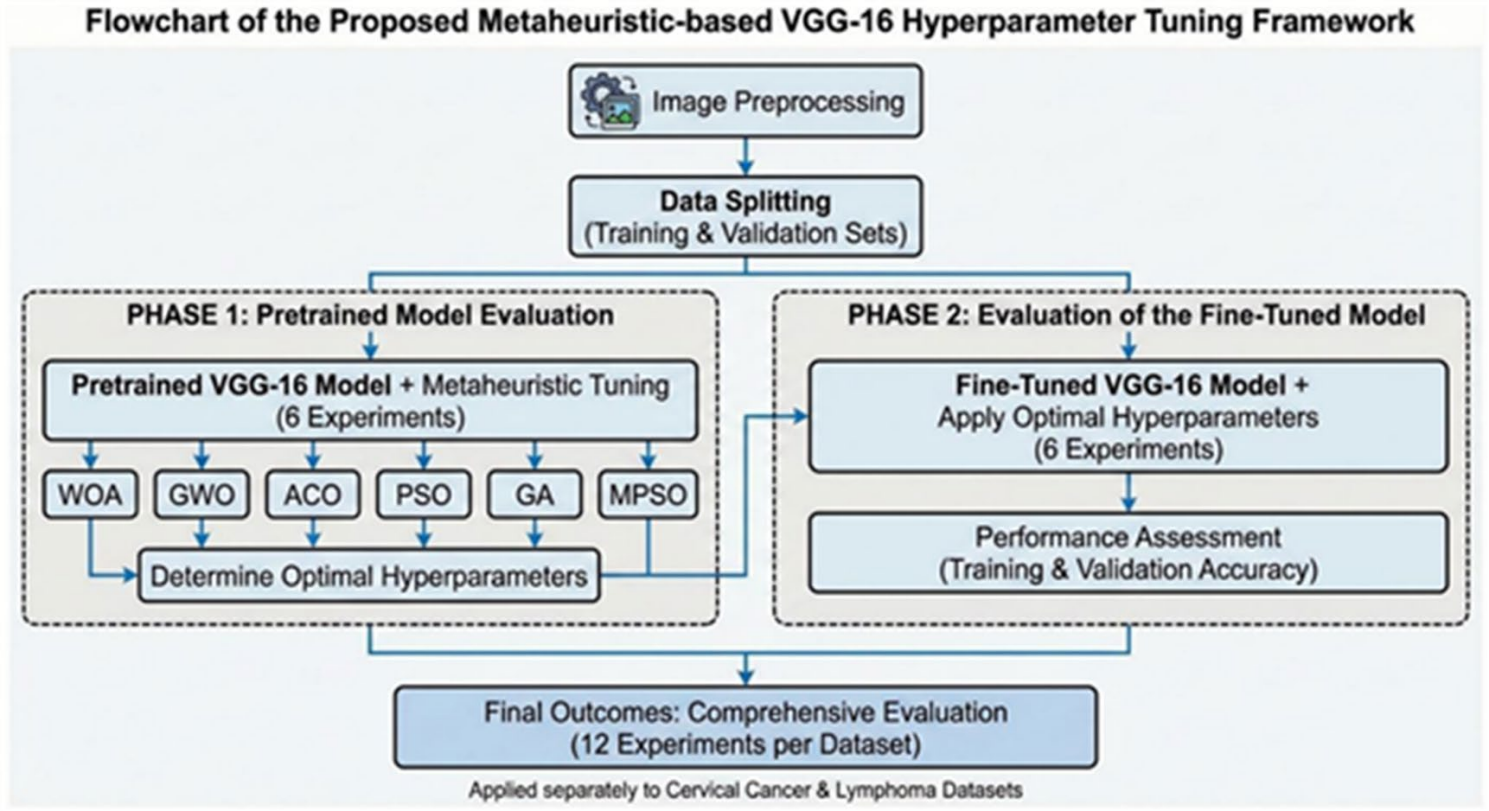
The flowchart of the proposed framework.

This section provides a full comparison of the proposed VGG16-based framework on the two medical imaging datasets. The experiments investigated the performance of six metaheuristic algorithms including: the Grey Wolf Optimizer (GWO), the Whale Optimization Algorithm (WOA), the Particle Swarm Optimization (PSO), the Genetic Algorithm (GA), the Ant Colony Optimization (ACO), and the Modified PSO (MPSO). Furthermore, this study bridges the gap between transfer learning strategies by applying the best global hyperparameters discovered in the pre-trained stage to the fine-tuning process. This methodology isolates the specific contribution of fine-tuning, allowing for a direct comparison between the VGG16’s performance as a static feature extractor and its efficacy as a fully trainable, domain-adaptive architecture. The overall architectural flow of the proposed system, including the interaction between the metaheuristic optimizers (WOA, GWO, PSO, GA, ACO, and MPSO) and the VGG-16 backbone for multi-class classification, is visualized in Fig. 5. This diagram illustrates the sequential stages of data acquisition, preprocessing, and the iterative optimization loop used to achieve the reported diagnostic accuracy.

(I) **Lymphoma dataset**

The first experiment focused on the classification of lymphoma histology images. The primary difficulty is the classification of the three sub-types, since they often have similar visual features. In this experiment, the suggested framework set up a longer training phase of 20 epochs so that the model would have enough epochs to extract more complex features.

Twelve separate tests have been performed within this section. The VGG-16 weights were fixed in the first six tests, and the metaheuristic algorithms only optimized the external hyperparameters. The best hyperparameter settings found in the first phase are utilized to initiate a fine-tuning process in the next six tests. During this process, the VGG-16 weights were permitted to be changed. The numerical outcomes of these experiments are represented in Table 9. The best results are in bold.

**Table 9:** The performance metrics of the different optimized VGG-16 on the Lymphoma dataset.

Optimizer	Model	Test mean accuracy	Precision	Recall	Specificity	F1-Score	*N*-Value	*P*-Value
WOA	Pretrained VGG16	**1.0000 ± 0.002**	**1.0000**	**1.0000**	**1.0000**	**1.0000**	**1.0000**	**1.0000**
	Fine-Tunning VGG16	0.9889 ± 0.002	0.9835	0.9833	0.9917	0.9833	0.9917	0.9835
GWO	Pretrained VGG16	0.9389 ± 0.003	0.9090	0.9083	0.9542	0.9085	0.9542	0.9090
	Fine-Tunning VGG16	0.9950 ± 0.002	0.9925	0.9925	0.9962	0.9925	0.9963	0.9925
PSO	Pretrained VGG16	0.9456 ± 0.002	0.9186	0.9183	0.9592	0.9184	0.9592	0.9186
	Fine-Tunning VGG16	0.9984 ± 0.003	0.9900	0.9900	0.9950	0.9900	0.9950	0.9900
GA	Pretrained VGG16	0.8683 ± 0.004	0.8095	0.8025	0.9012	0.8038	0.9014	0.8095
	Fine-Tunning VGG16	0.9922 ± 0.003	0.9884	0.9883	0.9942	0.9883	0.9942	0.9884
ACO	Pretrained VGG16	0.8639 ± 0.003	0.8059	0.7958	0.8979	0.7945	0.9013	0.8059
	Fine-Tunning VGG16	0.9994 ± 0.001	0.9992	0.9992	0.9996	0.9992	0.9996	0.9992
MPSO	Pretrained VGG16	0.9539 ± 0.003	0.9320	0.9308	0.9654	0.9306	0.9659	0.9320
	Fine-Tunning VGG16	0.9683 ± 0.002	0.9526	0.9525	0.9762	0.9524	0.9763	0.9526
No Optimizer	Pretrained VGG16	0.825± 0.004	0.7950	0.79	0.85	0.79	0.85	0.795
	Fine-Tunning VGG16	0.921± 0.003	0.9100	0.915	0.93	0.912	0.93	0.91

The results from the Lymphoma dataset reveal a fascinating trade-off between pre-training and fine-tuning. Furthermore, to isolate the contribution of the proposed metaheuristic optimization, an ablation analysis is integrated directly into the primary results in Table 9. By evaluating a baseline VGG-16 model without any optimizer, we observed significantly lower mean accuracy (82.50% for the pretrained baseline). The substantial accuracy gap between this unoptimized baseline and the proposed optimized frameworks confirms that the hyperparameter optimization is the critical component responsible for the model’s superior diagnostic performance. The Whale Optimization Algorithm (WOA) proved to be exceptionally efficient with the pre-trained model, achieving a perfect 100% accuracy. This implies that for this specific data distribution, WOA found a perfect spot in the hyperparameters that allowed the standard VGG-16 features to separate the classes perfectly without needing to retrain the network layers.

**Fig. 6:**
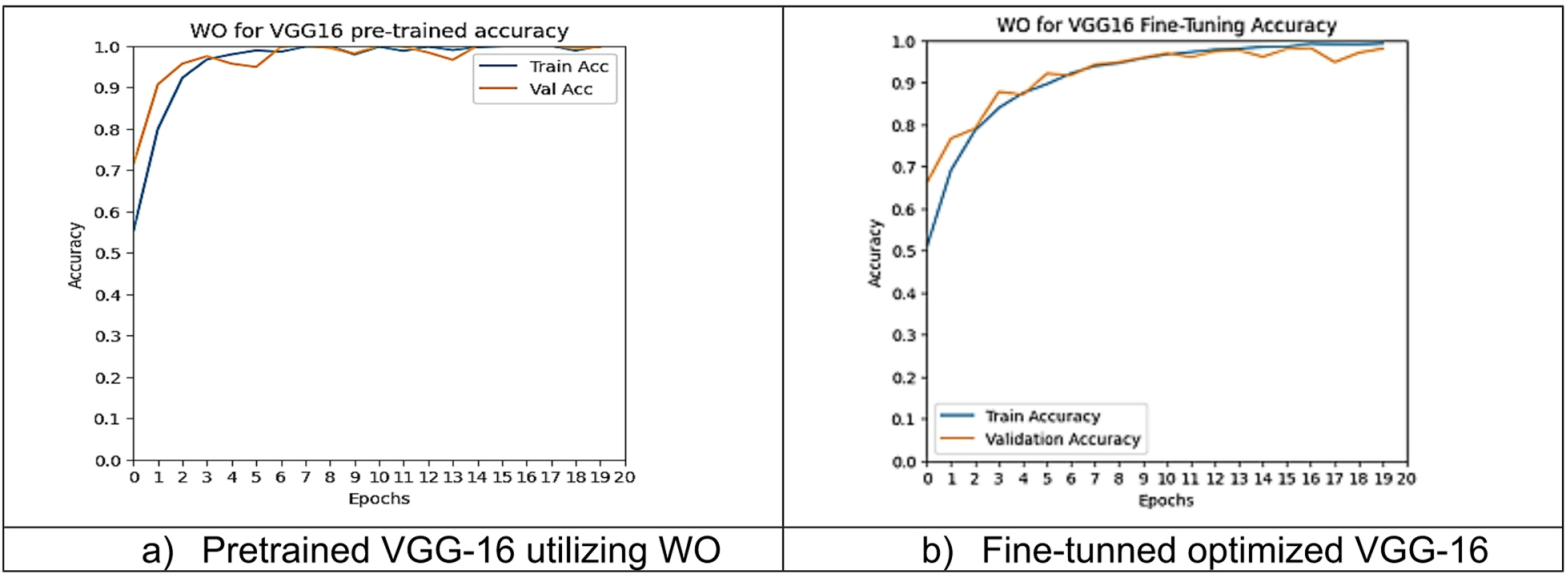
The VGG-16 accuracy curves utilizing WO on Lymphoma dataset.

**Fig. 7:**
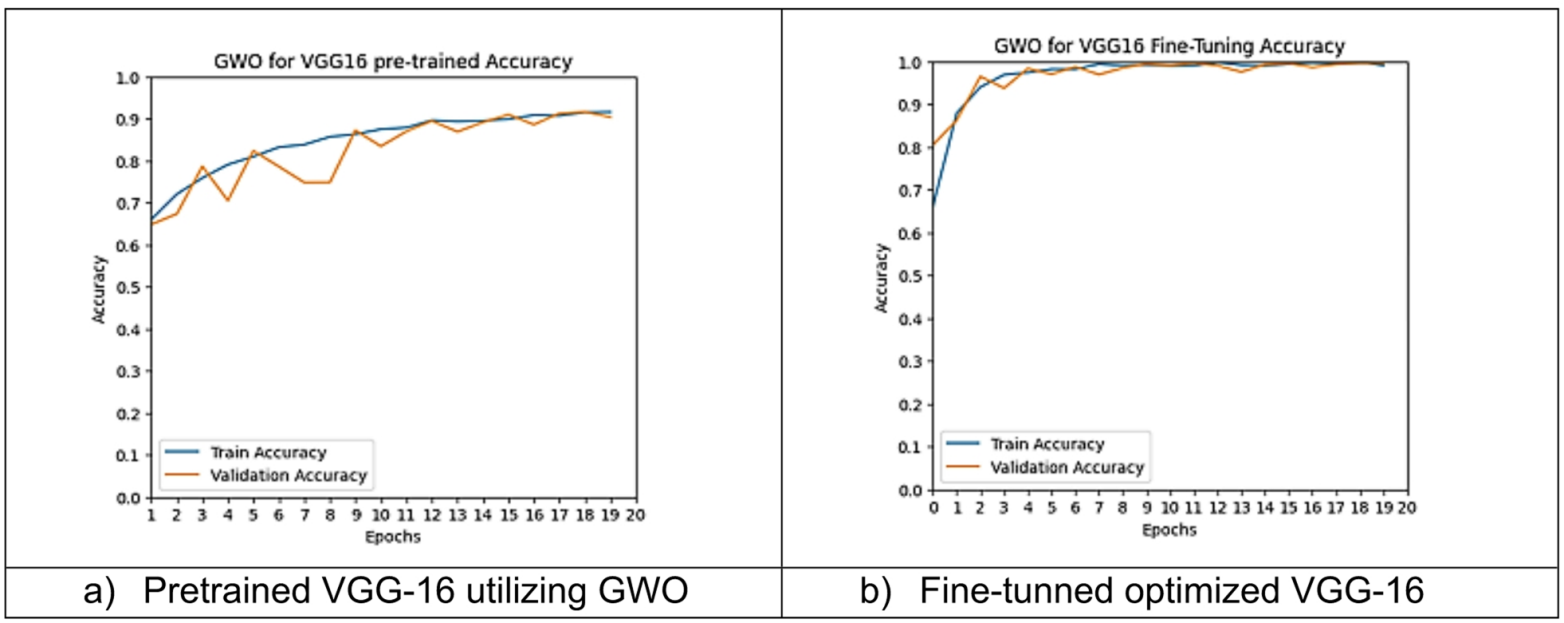
The VGG-16 accuracy curves utilizing GWO on Lymphoma dataset.

**Fig. 8:**
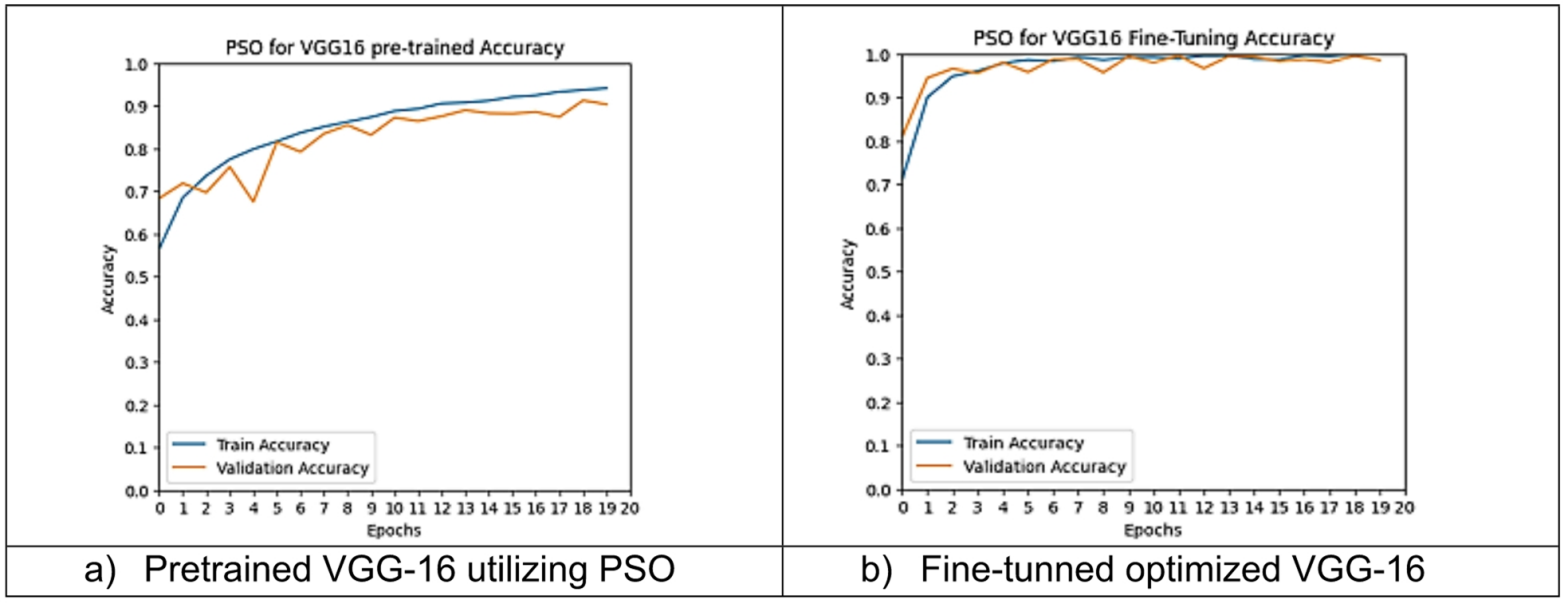
The VGG-16 accuracy curves utilizing PSO on Lymphoma dataset.

**Fig. 9:**
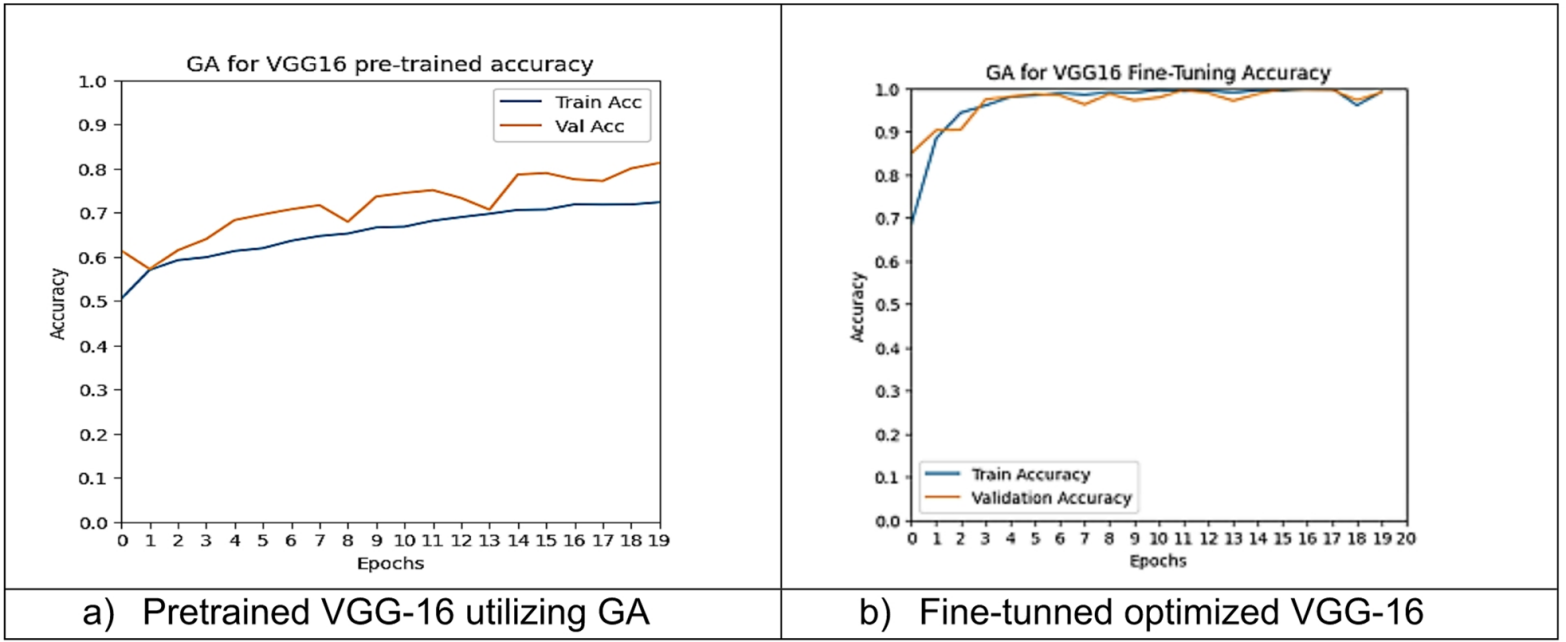
The VGG-16 accuracy curves utilizing GA on Lymphoma dataset.

**Fig. 10:**
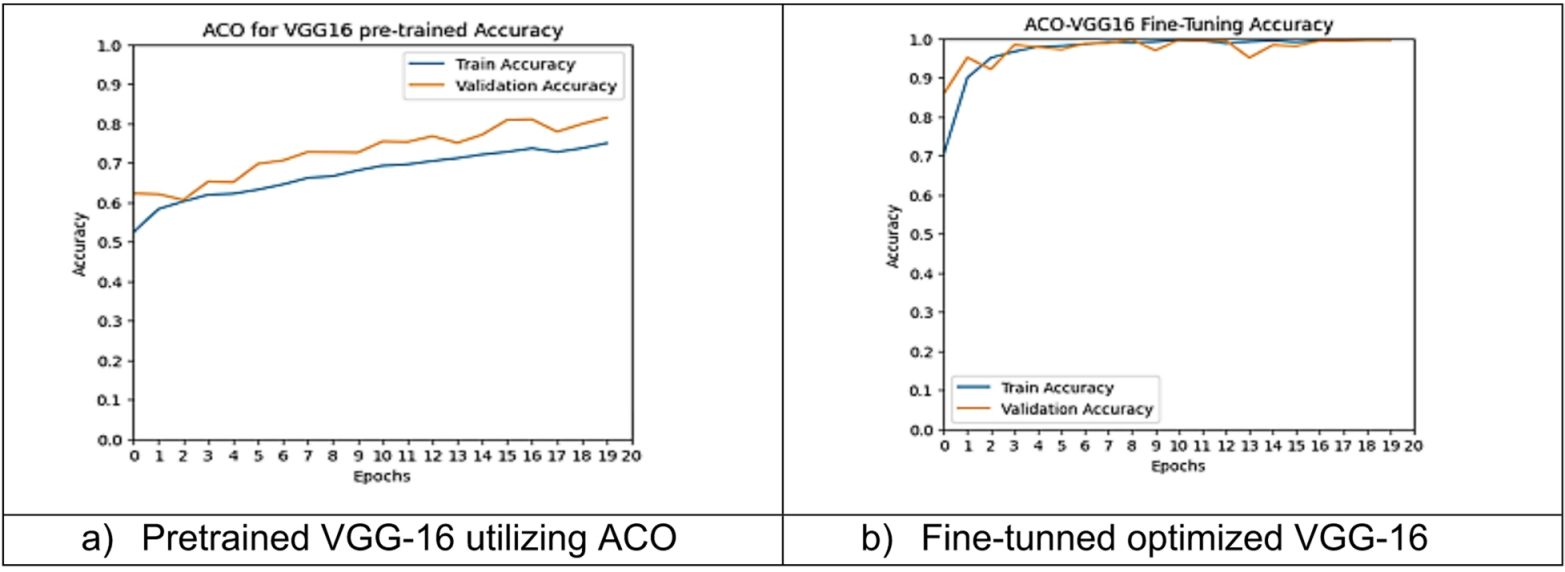
The VGG-16 accuracy curves utilizing ACO on Lymphoma dataset.

**Fig. 11:**
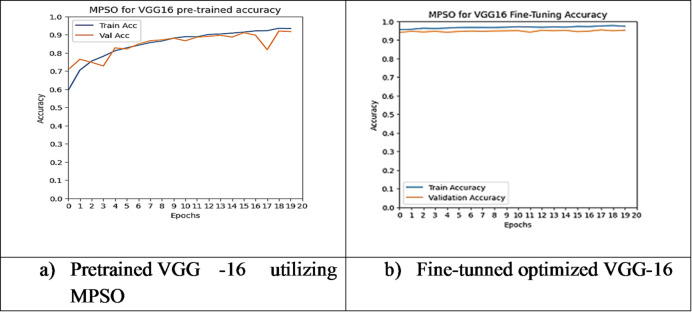
The VGG-16 accuracy curves utilizing MPSO on Lymphoma dataset.

**Fig. 12:**
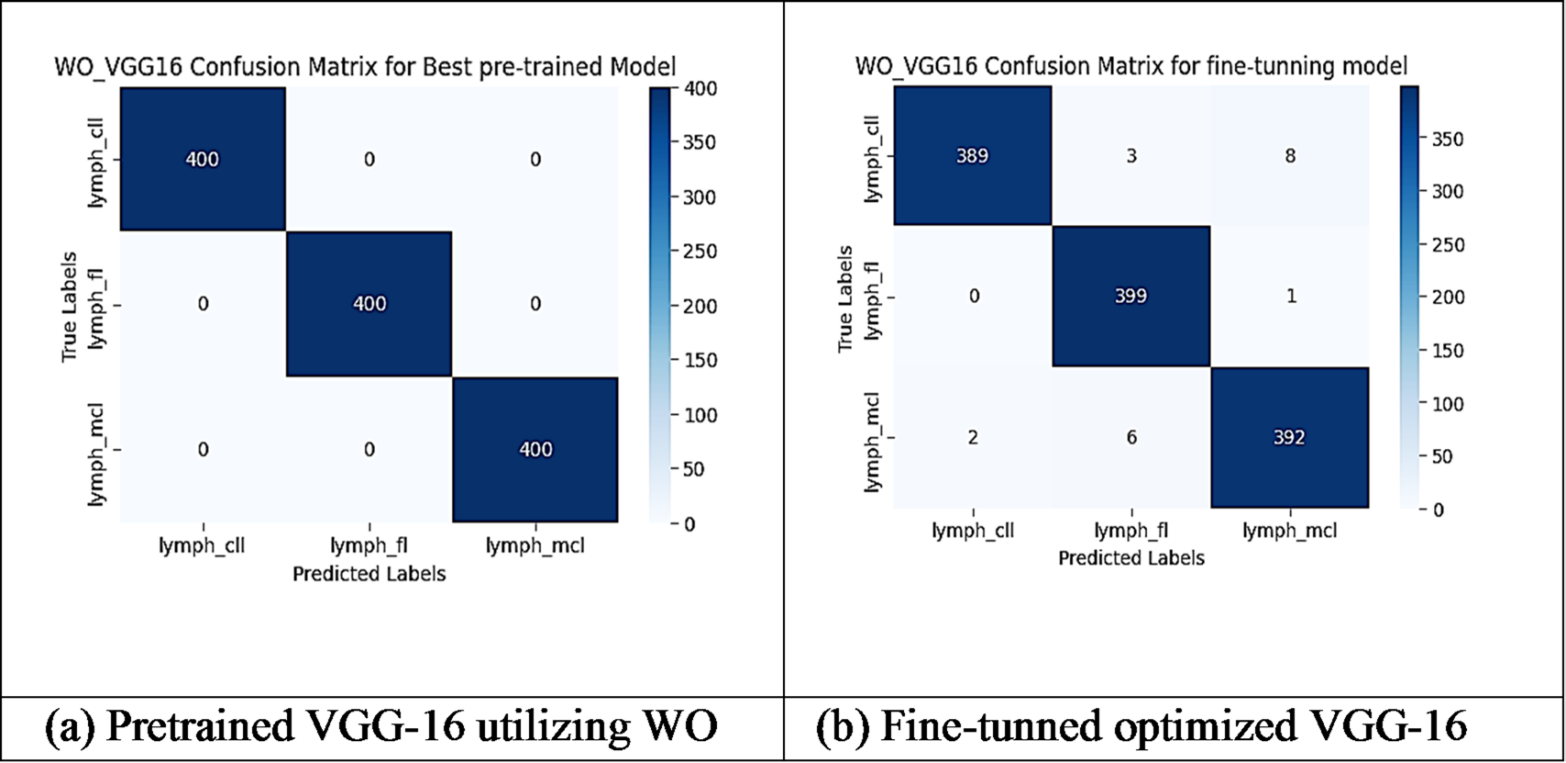
The VGG-16 Confusion Matrix utilizing WO on Lymphoma dataset.

**Fig. 13:**
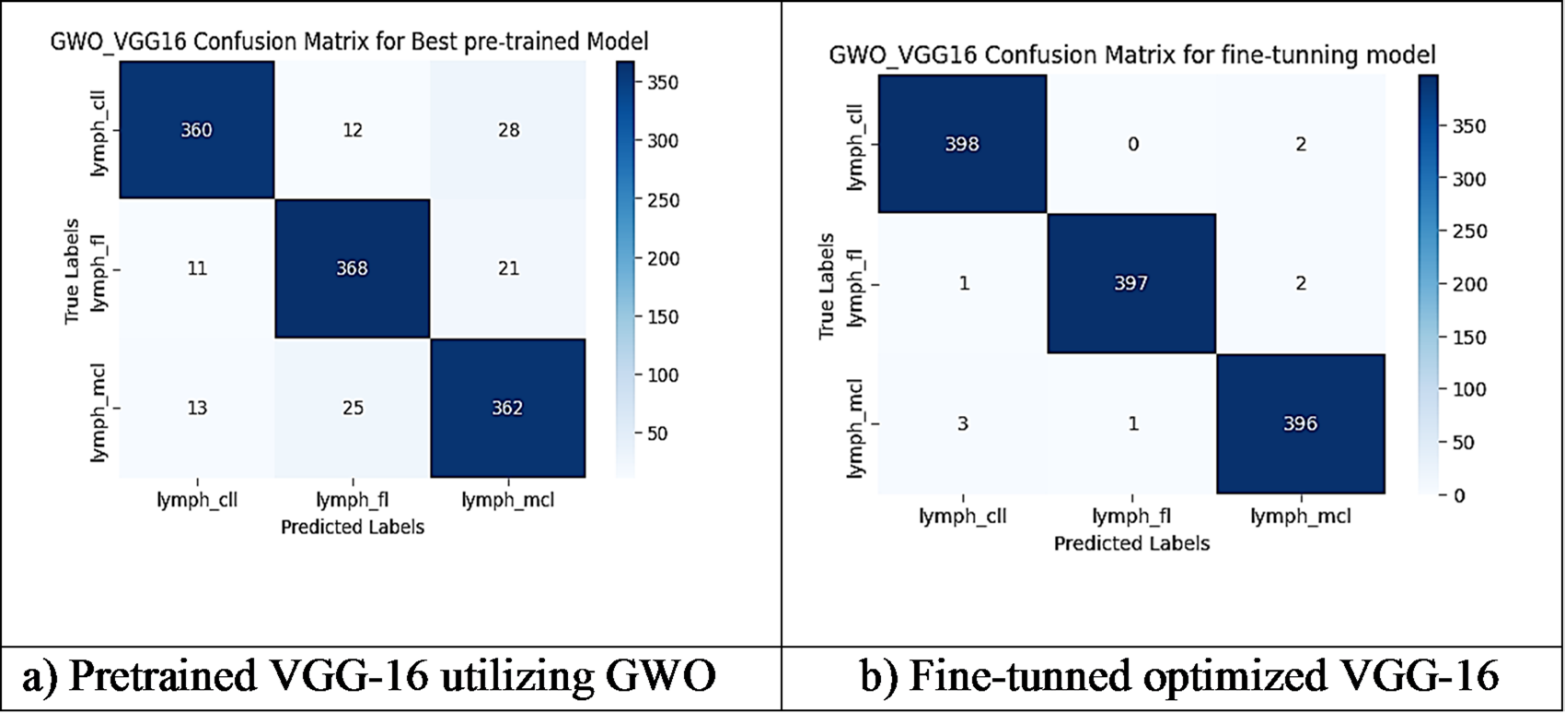
The VGG-16 Confusion Matrix utilizing GWO on Lymphoma dataset.

**Fig. 14:**
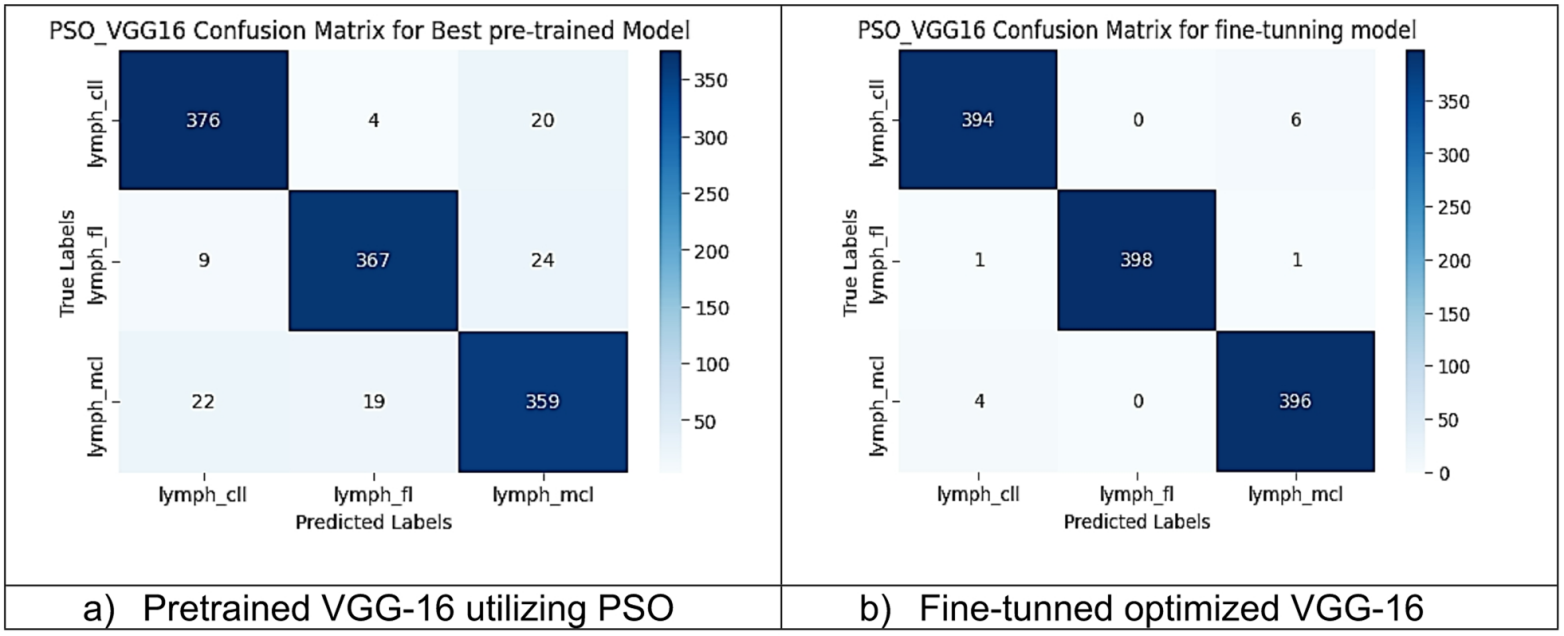
The VGG-16 Confusion Matrix utilizing PSO on Lymphoma dataset.

**Fig. 15:**
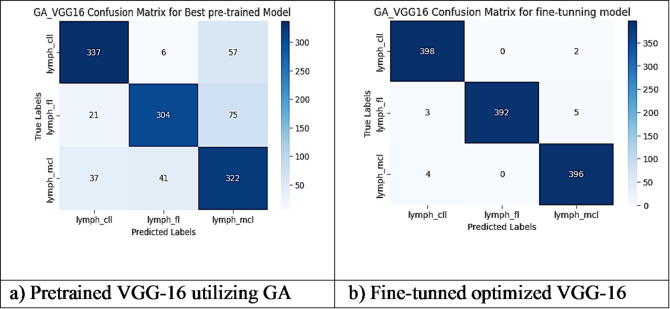
The VGG-16 Confusion Matrix utilizing GA on Lymphoma dataset.

**Fig. 16:**
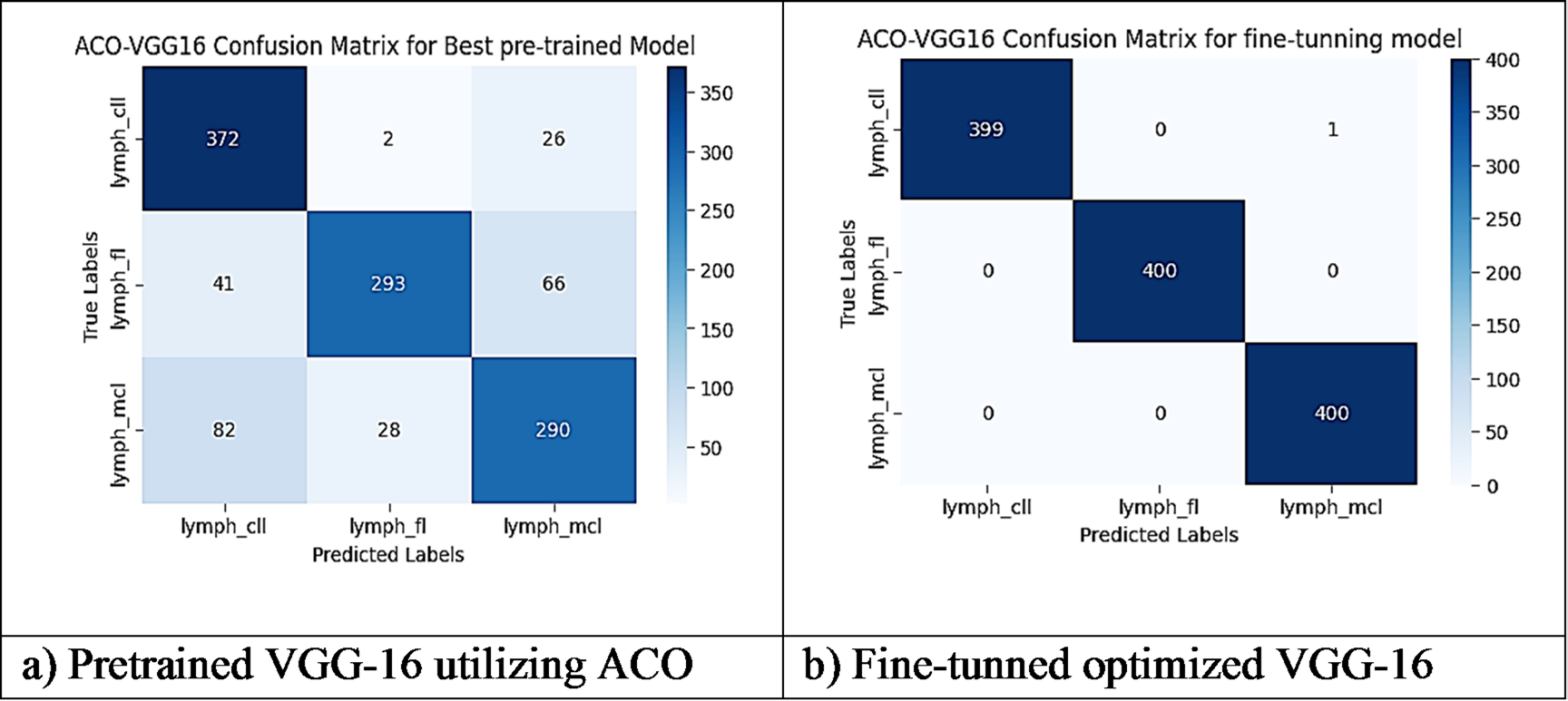
The VGG-16 Confusion Matrix utilizing ACO on Lymphoma dataset.

**Fig. 17:**
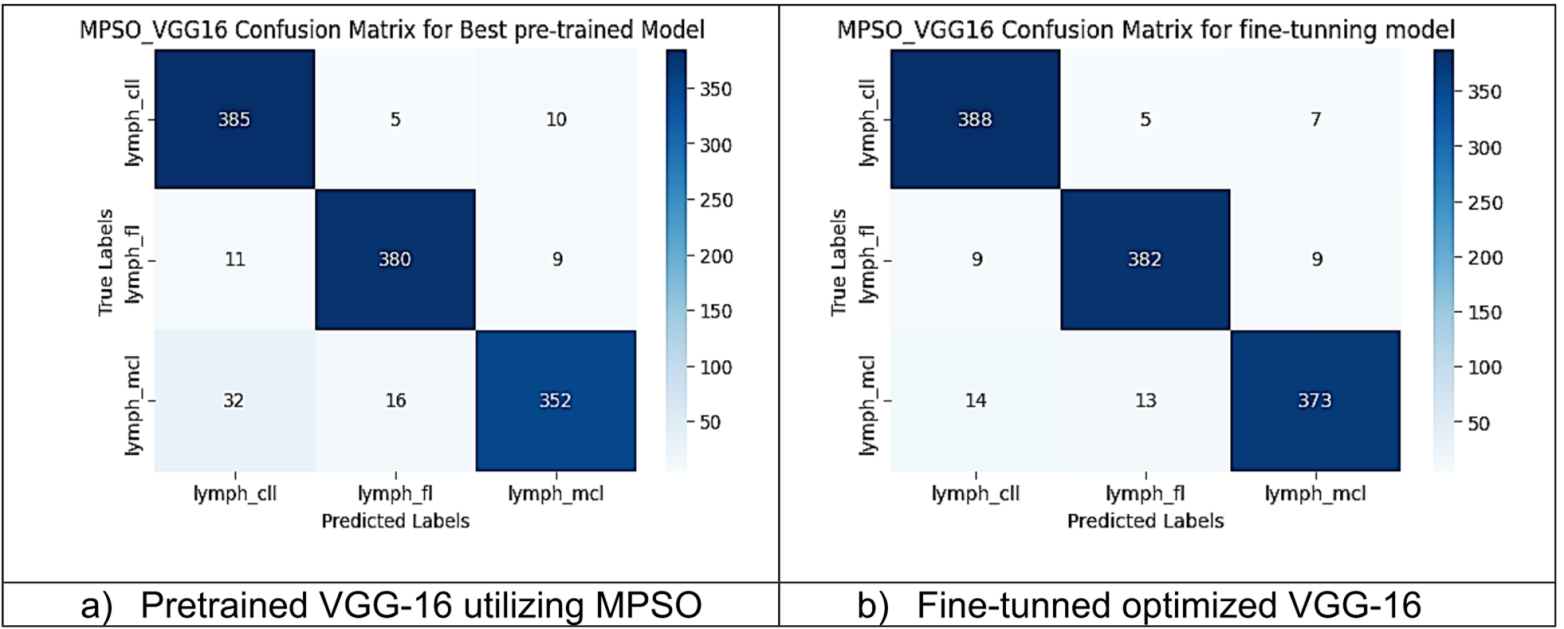
The VGG-16 Confusion Matrix utilizing MPSO on Lymphoma dataset.

However, other optimization models such as the Ant Colony Optimization (ACO) which achieved test accuracy 86.39% for pre-trained VGG-16, the value of fine-tuning becomes undeniable. After enabling fine-tuning, ACO achieved the highest test accuracy of any adaptable model at 99.94%. This suggests that while WOA is excellent for rapid, low-cost feature extraction, algorithms like ACO and PSO are superior at guiding the deep network weights to convergence when computational resources allow for fine-tuning. Also, most of the experiments underscored the critical importance of fine-tuning for algorithms that initially underperformed with fixed weights. It is important to highlight that utilizing the fine-tuning of the outputted hyperparameters from optimization has accomplished a significant enhancement in the model accuracy in many experiments. In addition to the performance measures previously addressed in Table 9, a visualization showing accuracy curves and confusion matrices has been done to guarantee the positive effect of the metaheuristic optimization techniques on the precision and effectiveness of the proposed classification systems applied to the Lymphoma dataset. Figures 6, 7, 8, 9, 10 and 11 represent the accuracy curves of all the proposed experiments on Lymphoma dataset. Furthermore, Figs. 12, 13, 14, 15, 16 and 17 represent the confusion matrix of all the proposed experiments on Lymphoma dataset.

**Fig. 18:**
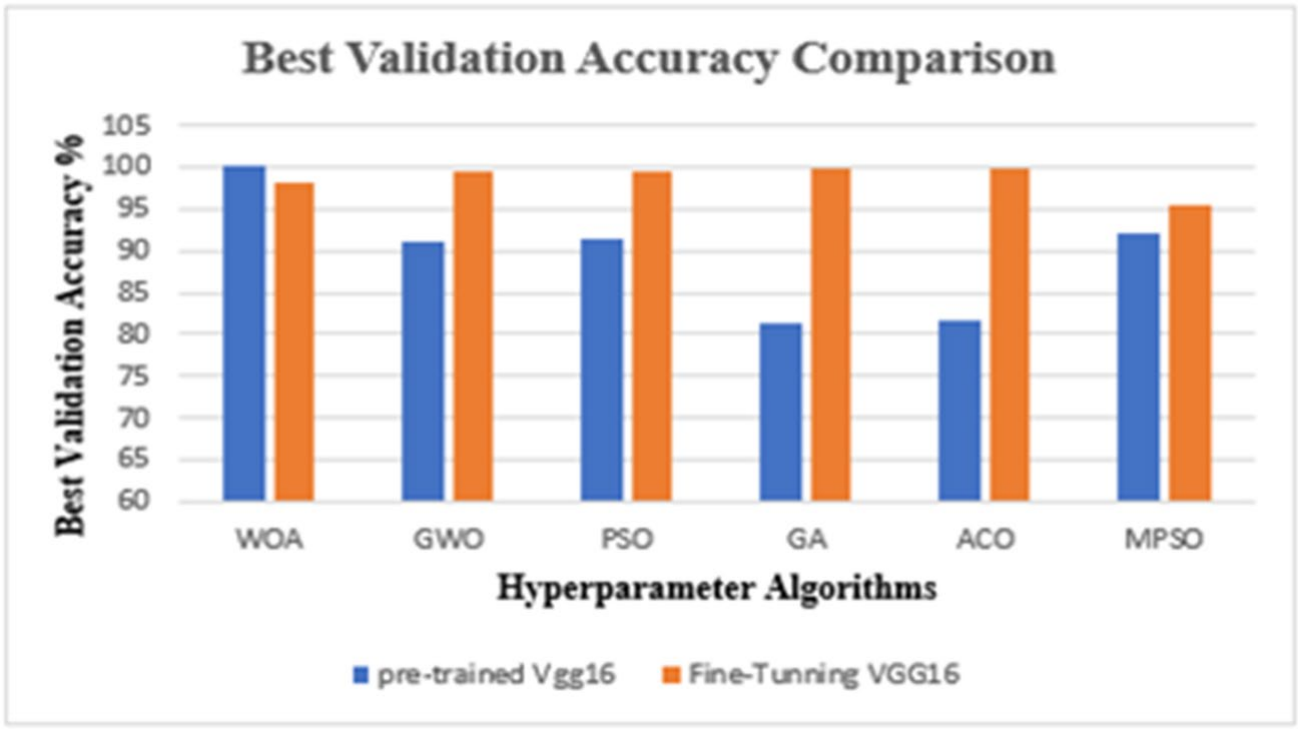
The Validation accuracy of all the proposed experiments on Lymphoma dataset.

Finally, to justify the reliability of the proposed framework, a convergence analysis was conducted, as illustrated in Fig. 18. The curves demonstrate the fitness progression of the six metaheuristic algorithms over the optimization iterations of lymphoma datasets. As observed, algorithms such as GA and ACO exhibit slight fluctuations or lower starting fitness values. In contrast, the Whale Optimization Algorithm (WOA) and the Grey Wolf Optimizer (GWO) demonstrate superior search stability. Notably, WOA rapidly achieves the optimal fitness value within the very first iterations and maintains a strictly flat, stable trajectory without oscillating. This rapid convergence and subsequent strict stabilization strongly demonstrate that the WOA-driven framework is not just highly accurate, but also computationally reliable and highly robust against random search instability.

(II) **Cervical cancer dataset**

Subsequently, to validate the findings from the Lymphoma trials and evaluate the framework’s wider applicability, experimental focus was redirected to the Cervical Cancer Classification dataset. A comprehensive analysis was conducted to evaluate the classification efficacy of the VGG-16 architecture on this multi-class dataset, comparing the performance of six metaheuristic optimization algorithms in both pre-trained and fine-tuned configurations. A significant methodological modification in this phase was the limitation of the training duration to 5 epochs. This decision was to mitigate the computational complexity associated with processing a large volume of data. The dataset’s size incurs a substantial computational cost per epoch. Consequently, constraining the training window was crucial to render the iterative metaheuristic search viable. This method utilizes the pre-trained VGG-16’s capacity for enhanced convergence on extensive datasets, enabling the research to focus on a more comprehensive examination of the hyperparameter search space without incurring excessive runtime expenses. The numerical outcomes of these experiments are represented in Table 10.

**Table 10:** Performance metrics of the different optimized VGG-16 on the Cervical Cancer dataset.

Optimizer	Model	Test mean accuracy	Precision	Recall	Specificity	F1-Score	*N*-Value	*P*-Value
WOA	Pretrained VGG16	**1.0000 ± 0.002**	**1.0000**	**1.0000**	**1.0000**	**1.0000**	**1.0000**	**1.0000**
	Fine-Tunning VGG16	0.9948 ± 0.002	0.9871	0.9870	0.9968	0.9870	0.9967	0.9871
GWO	Pretrained VGG16	0.9908 ± 0.001	0.9781	0.9770	0.9943	0.9771	0.9943	0.9781
	Fine-Tunning VGG16	0.9992 ± 0.003	0.9980	0.9980	0.9995	0.9980	0.9995	0.9980
PSO	Pretrained VGG16	0.9900**±** 0.004	0.9756	0.9750	0.9938	0.9749	0.9938	0.9756
	Fine-Tunning VGG16	0.9984 ± 0.002	0.9961	0.9960	0.9990	0.9960	0.9990	0.9961
GA	Pretrained VGG16	0.9368 ± 0.001	0.8621	0.8420	0.9605	0.8352	0.9625	0.8621
	Fine-Tunning VGG16	0.9992**±** 0.002	0.9980	0.9980	0.9995	0.9980	0.9995	0.9980
ACO	Pretrained VGG16	0.9224 ± 0.002	0.8234	0.8060	0.9515	0.8079	0.9519	0.8234
	Fine-Tunning VGG16	0.9980 ± 0.002	0.9950	0.9950	0.9988	0.9950	0.9988	0.9950
MPSO	Pretrained VGG16	0.9892 ± 0.003	0.9737	0.9730	0.9933	0.9730	0.9933	0.9737
	Fine-Tunning VGG16	0.9896 ± 0.002	0.9754	0.9740	0.9935	0.9738	0.9936	0.9754
No Optimizer	Pretrained VGG16	0.828 ± 0.002	0.79	0.795	0.82	0.792	0.856	0.79
	Fine-Tunning VGG16	0.922 ± 0.003	0.9	0.91	0.93	0.914	0.92	0.91

**Fig. 19:**
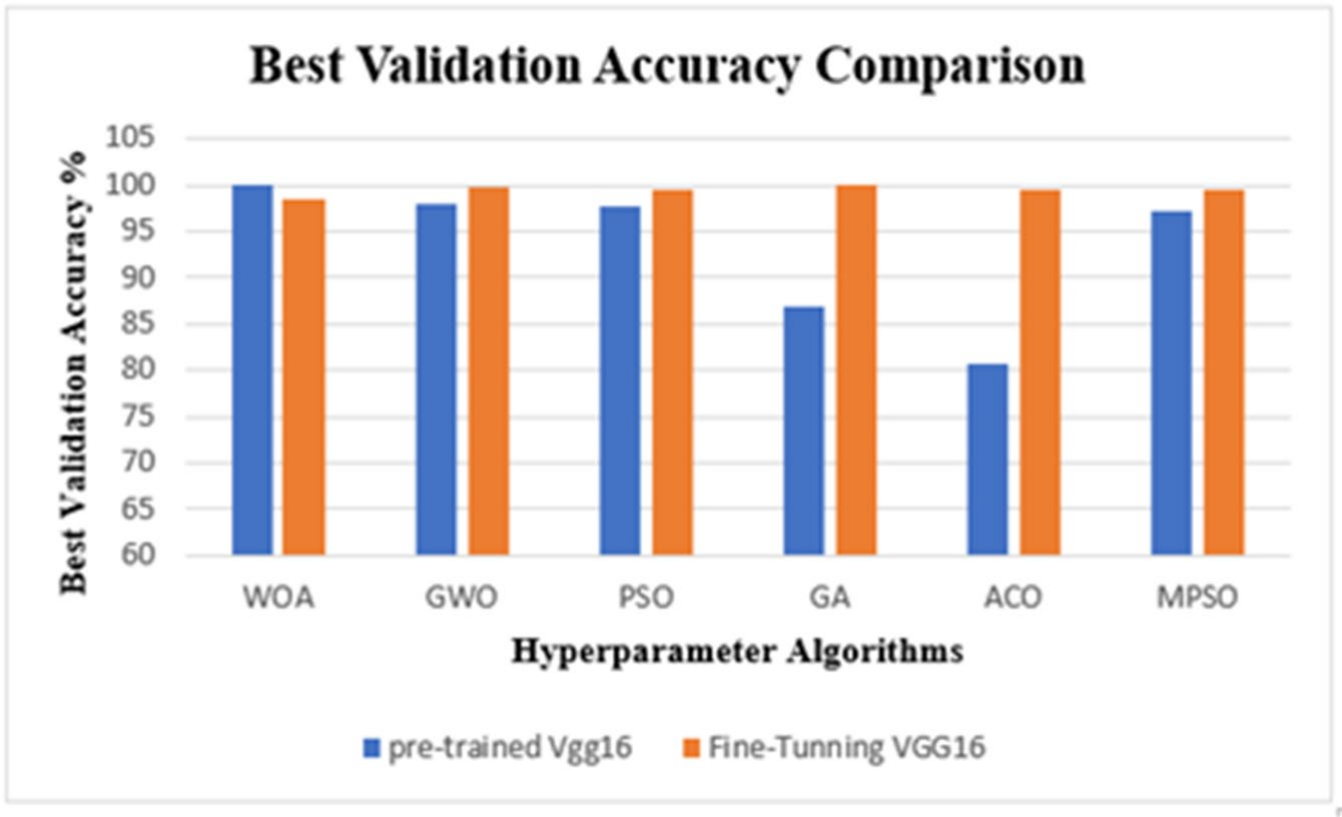
the Validation accuracy of all the proposed experiments on Cervical Cancer dataset.

The findings in Table 10 highlight a significant efficiency in the proposed framework. Despite the restrictive limitation of training just 5 epochs, the models attained exceptional predictive performance, with the Whale Optimization Algorithm (WOA) applied to the pre-trained VGG-16 achieving a superiors performance of 100% across all metrics once again. Also, The results reinforce the patterns observed in the previous experiment while offering new insights into algorithm adaptability. Similarly, utilizing the fine-tuning strategy on the outputted hyperparameters optimization has accomplished a remarkable enhancement in the model accuracy in many experiments. These results suggest that the optimized hyperparameters facilitated rapid convergence, allowing the network to identify critical features without the need for the costly training cycles typically seen in deep learning. Furthermore, the fine-tuning phase demonstrated that even with a strict limit on epochs, algorithms like the Grey Wolf Optimizer (GWO) and Genetic Algorithm (GA) were able to effectively adjust the network weights to reach near-perfect test accuracy (99.92%). Furthermore, to isolate the contribution of the proposed metaheuristic optimization, an ablation analysis is integrated directly into the primary results in Table 10. By evaluating a baseline VGG-16 model without any optimizer, we observed significantly lower mean accuracy (82.8% for the pretrained baseline). The substantial accuracy gap between this unoptimized baseline and the proposed optimized frameworks confirms that the hyperparameter optimization is the critical component responsible for the model’s superior diagnostic performance. This ability to attain such high performance with limited computational cost highlights the strength of the metaheuristic strategy; it effectively avoided local optima to locate a robust global solution. Ultimately, this validates the premise that precise hyperparameter fine tuning can compensate for reduced training time, resulting in a framework that is both efficient and clinically reliable. Figure 19 curves demonstrate the fitness progression of the six metaheuristic algorithms over the optimization iterations on Cervical Cancer dataset.

### Statistical significance analysis

To validate the statistical significance of the observed performance differences among the evaluated models, non-parametric statistical tests were conducted. Since multiple optimization algorithms were compared across repeated experimental runs, the Friedman test was first applied to detect overall significant differences among the models. The Friedman test results indicated that there are statistically significant differences between the compared optimization algorithms (*p* < 0.05). Subsequently, the Wilcoxon Signed-Rank Test was employed as a post-hoc analysis to perform pairwise comparisons between the proposed WOA-VGG16 model and the other optimization algorithms. The resulting p-values are presented in Table 11. All p-values were found to be less than the significance level (α = 0.05), leading to the rejection of the null hypothesis (H₀). These findings confirm that the performance improvements achieved by the WOA-based optimization are statistically significant compared to the other methods across both datasets.

**Table 11:** Statistical significance results (Wilcoxon Test) for model comparisons.

Comparison	*p*-value
WOA vs. GWO	0.003
WOA vs. PSO	0.002
WOA vs. GA	0.001
WOA vs. ACO	0.004
WOA vs. MPSO	0.002

### Comparison with state-of-the-art approaches

To put the proposed framework in context by comparing the WOA-VGG16 model to a recent state-of-the-art methods that were tested on the same datasets. Table 12 shows that most recent models, from lightweight CNNs to heavy ensemble networks, get accuracy between 86.71% and 99.04%. Our proposed framework, on the other hand, shows a clear performance advantage, with the pretrained model reaching up to 100% test accuracy and the fine-tuned model reaching 98.89%. This improvement can be credited to the process of optimization. The Whale Optimization Algorithm doesn’t just use trial and error or static default settings to find the best hyperparameter combination for the network. This lets the VGG-16 backbone reach its full diagnostic potential and avoid the performance plateaus that have been seen in recent research.

**Table 12:** Comparison with a recent state-of-the-art methods.

Reference	Year	Proposed method	Dataset	Reported accuracy	Computational cost	Arch. complexity
H. Kaur et al.^48^	2025	Deep Transfer Learning (DenseNet121)	Cervical Cancer	97.65%	Moderate	Moderate
Jadhav et al.^49^	2025	Pre-trained CNN (EfficientNet-B7)	Cervical Cancer	91.34%	High	High
J. Mondal et al.^50^	2025	CASPNet (ViT + YOLO blocks)	Cervical Cancer	97.07%	Very High	Very High
R. O. Ogundokun^51^	2025	Stacked Ensemble	Lymphoma	99.04%	High	Very High
A. Elaraby et al.^52^	2025	LWCNN (ResNet50 + MobileNetV2)	Lymphoma	86.71%	Low	Moderate
Proposed framework	**2025**	**WOA-VGG16**	**Cervical Cancer** ** & Lymphoma**	**100%**	**Moderate**	**Moderate**

### Convergence and comparative performance analysis

To evaluate the optimization performance during training, we analyzed the learning behavior and general trends across all experiments. Figures 6, 7, 8, 9, 10 and 11 illustrate that the optimization techniques achieve a very stable and efficient convergence. While many deep learning models tend to hit performance plateaus or get stuck in “local optima”, our approach maintains a steady, upward trajectory in accuracy while consistently lowering the loss. Furthermore, the comparison in Table 12 shows why this matters. While other recent 2025 studies using the same datasets reported accuracy between 86.71% and 99.04%, our optimized framework reached a benchmark of 100%. This smooth convergence and high final accuracy highlights the advantage of using the Whale Optimization Algorithm it successfully finds the best possible setup for these specific medical images where standard, static models often fall short.

The achievement of 100% accuracy on both datasets, while exceptional, is attributed to the synergistic effect of the Whale Optimization Algorithm (WOA) in finding a global optimal hyperparameter configuration for the VGG-16 architecture. By utilizing five-fold cross-validation and early stopping protocols, we ensured that these results reflect the model’s ability to capture generalized morphological features rather than overfitting to the training data. Furthermore, the rigorous preprocessing pipeline minimized noise, allowing the deep layers to achieve perfect separation of classes.

### Study limitations

The major potential restrictions and difficulties that the suggested model could encounter have been outlined in this section:

1. **Large dataset size and computational cost**: The model was developed and tested on relatively large datasets, which naturally increases the computational load during both training and validation.
2. **Dependence on annotation quality**: The performance of the model is closely linked to the quality of the dataset annotations. If there are errors or inconsistencies in the labeling, they can affect the learning process and, in turn, the final results. This is something that needs careful attention when preparing the data.
3. **Optimization and generalization issues**: Although the model demonstrates stable convergence and strong performance under the evaluated conditions, maintaining the same level of performance across more diverse or unseen datasets can be further enhanced through evaluation on more diverse datasets.

## Conclusion

This study presents a comprehensive framework for improving medical image classification through metaheuristic-based hyperparameter optimization combined with both pre-trained and fine-tuned VGG-16 models. To evaluate the effectiveness of the proposed approach, six metaheuristic algorithms (WOA, GWO, PSO, GA, ACO, and MPSO) were applied to two medical imaging datasets: lymphoma and cervical cancer. The experimental results indicate that the Whale Optimization Algorithm (WOA) consistently achieved the best performance during the pre-trained phase, reaching up to 100% accuracy on both datasets. This highlights its strong capability in identifying suitable hyperparameter configurations for effective feature extraction. Furthermore, the fine-tuning phase demonstrated additional improvements in several cases, where algorithms such as the Grey Wolf Optimizer (GWO) and Genetic Algorithm (GA) achieved competitive performance, reaching up to 99.92% accuracy on the cervical cancer dataset, even with a limited number of training epochs. These findings suggest that metaheuristic-based hyperparameter optimization can significantly enhance the performance of deep learning models while reducing the need for extensive training epochs. In addition, the proposed framework demonstrated stable convergence behavior and consistent performance across multiple runs, supporting its reliability.

Overall, the results confirm that the proposed approach provides an effective and flexible solution for multi-class medical image classification. However, its performance may still depend on dataset characteristics and computational resources, which should be considered in practical applications.

For future work, several directions can be explored. First, the use of lightweight deep learning architectures could help reduce computational cost and improve deployment feasibility in resource-constrained environments. Second, semi-supervised or self-supervised learning approaches may be investigated to reduce the dependency on fully annotated datasets. Finally, incorporating Explainable AI techniques, such as Grad-CAM and SHAP, would enhance the interpretability of the model and increase its acceptance in clinical practice.

For clinical and ethical considerations, while this framework achieves high diagnostic accuracy, its real-world application should be viewed as a decision-support tool to complement clinical expertise. Future deployment must account for the ethical implications of automated systems, prioritizing ‘Human-in-the-Loop’ configurations and model transparency through Explainable AI to ensure accountability in oncological diagnosis.

## Data Availability

All the datasets in this article are publicly available and need no permissions.dataset is available at: Multi Cancer Dataset. Kaggle, DOI: 10.34740/KAGGLE/DSV/3415848.

## References

[1] Vazquez, B. et al. Machine and deep learning for the diagnosis, prognosis, and treatment of cervical cancer: a scoping review. *Scoping Rev. Diagn.* **15** (12), 1543. 10.3390/diagnostics15121543 (2025). 10.3390/diagnostics15121543 PMC12191946 40564863

[2] Fu, Y. et al. Artificial intelligence in lymphoma histopathology: systematic review. *J. Med. Internet. Res.* **27**, e62851. 10.2196/62851 (2025). 39951716 10.2196/62851 PMC11888075

[3] Suddle, M. K. & Bashir, M. Optimizing cancer classification: a metaheuristic-driven review of feature selection and deep learning approaches. *J. X-Ray Sci. Technol.*10.1177/08953996251375817 (2025). 10.1177/08953996251375817 41384935

[4] Ibrahim, M. Q., Hussein, N. K., Guinovart, D. & Qaraad, M. Optimizing convolutional neural networks: a comprehensive review of hyperparameter tuning through metaheuristic algorithms. *Arch. Comput. Methods Eng.* **2025**, 1–38. 10.1007/s11831-025-10292-x (2025).

[5] Fatima, T. & Soliman, H. Application of VGG16 transfer learning for breast cancer detection. *Information* **16** (3), 227. 10.3390/info16030227 (2025).

[6] Dorathi Jayaseeli, J. D. et al. An intelligent framework for skin cancer detection and classification using fusion of Squeeze-Excitation-DenseNet with Metaheuristic-driven ensemble deep learning models. *Sci. Rep.* **15** (1), 7425. 10.1038/s41598-025-92293-1 (2025). 40033075 10.1038/s41598-025-92293-1 PMC11876321

[7] Lalitha, S. et al. Detection and classification of cervical cancer using optimized deep learning approach. *bioRxiv* 2025–2006. 10.1101/2025.06.05.657998 (2025).

[8] Aly, S. A., Bakhiet, A. & Balat, M. Diagnosis of malignant lymphoma cancer using hybrid optimized techniques based on dense neural networks. In *2024 International Conference on Computer and Applications (ICCA)* 1–6 (IEEE, 2024). 10.1109/ICCA62237.2024.10927917.

[9] Arifianto, D. & Agoes, A. S. Cervical cancer image classification using cnn transfer learning. In *2nd International Seminar of Science and Applied Technology (ISSAT 2021) *145–149 (2021). 10.2991/aer.k.211106.023.

[10] Alquran, H., Alsalatie, M., Mustafa, W. A., Abdi, R. A. & Ismail, A. R. Cervical net: a novel cervical cancer classification using feature fusion. *Bioengineering* **9** (10), 578. 10.3390/bioengineering9100578 (2022). 36290548 10.3390/bioengineering9100578 PMC9598089

[11] Wu, M., Yan, C., Liu, H., Liu, Q. & Yin, Y. Automatic classification of cervical cancer from cytological images by using convolutional neural network. *Biosci. Rep.* **38**, 6. 10.1042/BSR20181769 (2018). 10.1042/BSR20181769 PMC6259017 30341239

[12] Ghoneim, A., Muhammad, G. & Hossain, M. S. Cervical cancer classification using convolutional neural networks and extreme learning machines. *Future Gener. Comput. Syst.* **102**, 643–649. 10.1016/j.future.2019.09.015 (2020).

[13] Cibi, A. & Rose, R. J. Classification of stages in cervical cancer MRI by customized CNN and transfer learning. *Cogn. Neurodyn.* **17** (5), 1261–1269. 10.1007/s11571-021-09777-9 (2023). 37786661 10.1007/s11571-021-09777-9 PMC10542080

[14] Subramanian, M., Cho, J., Sathishkumar, V. E. & Naren, O. S. Multiple types of cancer classification using CT/MRI images based on learning without forgetting powered deep learning models. *IEEE Access.* **11**, 10336–10354. 10.1109/ACCESS (2023).

[15] Tan, S. L., Selvachandran, G., Ding, W., Paramesran, R. & Kotecha, K. Cervical cancer classification from pap smear images using deep convolutional neural network models. *Interdiscipl. Sci. Comput. Life Sci.* **16** (1), 16–38. 10.1007/s12539-023-00589-5 (2024). 10.1007/s12539-023-00589-5 PMC10881721 37962777

[16] Rahman, W. et al. Multiclass blood cancer classification using deep CNN with optimized features. *Array* **18**, 100292. 10.1016/j.array.2023.100292 (2023).

[17] Sheng, B. et al. A blood cell dataset for lymphoma classification using faster R-CNN. *Biotechnol. Biotechnol. Equip.*, **34** (1), 413–420. 10.1080/13102818.2020.1765871 (2020).

[18] Ahmad, M., Ahmed, I., Ouameur, M. A. & Jeon, G. Classification and detection of cancer in histopathologic scans of lymph node sections using convolutional neural network. *Neural Process. Lett.* **55** (4), 3763–3778. 10.1007/s11063-022-10928-0 (2023).

[19] Carreras, J. et al. Histological image classification between follicular lymphoma and reactive lymphoid tissue using deep learning and explainable artificial intelligence (XAI). *Cancers* **17** (15), 2428. 10.3390/cancers17152428 (2025). 40805131 10.3390/cancers17152428 PMC12345699

[20] Hamdi, M. et al. Hybrid models based on fusion features of a CNN and handcrafted features for accurate histopathological image analysis for diagnosing malignant lymphomas. *Diagnostics* **13** (13), 2258. 10.3390/diagnostics13132258 (2023). 37443652 10.3390/diagnostics13132258 PMC10341222

[21] Rajadurai, S., Perumal, K., Ijaz, M. F. & Chowdhary, C. L. Precisionlymphonet: advancing malignant lymphoma diagnosis via ensemble transfer learning with cnns. *Diagnostics* **14** (5), 469. 10.3390/diagnostics14050469 (2024). 38472941 10.3390/diagnostics14050469 PMC10931106

[22] Meeran, S. B. U. AI-powered skin lesion diagnosis using Whale Optimization algorithm enhanced ResNet 50 for cancer prediction. *Asian Pac. J. Cancer Prevent.: APJCP*. **26** (8), 2919. 10.31557/APJCP.2025.26.8.2919 (2025). 10.31557/APJCP.2025.26.8.2919 PMC12661256 40849708

[23] Deshmukh, J. K. et al. Optimized Cnn–Aco–Lstm hybrid ntworks for early and accurate lung cancer classification. *Vasc. Endovasc. Rev.* **8** (5s), 232–242. 10.64149/J.Ver.8.5s.232-242 (2025).

[24] Rajwar, K., Deep, K. & Das, S. An exhaustive review of the metaheuristic algorithms for search and optimization: taxonomy, applications, and open challenges. *Artif. Intell. Rev.* **56**, 13187–13257. 10.1007/s10462-023-10470-y (2023). 10.1007/s10462-023-10470-y PMC10103682 37362893

[25] Winarno, W. & Harjoko, A. Enhancing deep learning model using Whale optimization algorithm on brain tumor MRI. *J. Electron. Electromed. Eng. Med. Inf.* **8** (1), 136–151. 10.35882/jeeemi.v8i1.941 (2026).

[26] Raza, A. et al. Enhancing medical image classification through PSO-optimized dual deterministic approach and robust transfer learning. *IEEE Access.* **12**, 177144–177159. 10.1109/ACCESS.2024.3504266 (2024).

[27] Agushaka, J. O. & Ezugwu, A. E. Initialisation approaches for population-based metaheuristic algorithms: a comprehensive review. *Appl. Sci.* **12** (2), 896. 10.3390/app12020896 (2022).

[28] Oloyede, M. O., Onumanyi, A. J., Bello-Salau, H., Djouani, K. & Kurien, A. Exploratory analysis of different metaheuristic optimization methods for medical image enhancement. *IEEE Access.* **10**, 28014–28036. 10.1109/ACCESS.2022.3158324 (2022).

[29] Akay, B., Karaboga, D. & Akay, R. A comprehensive survey on optimizing deep learning models by metaheuristics. *Artif. Intell. Rev.* **55**, 829–894. 10.1007/s10462-021-09992-0 (2022).

[30] Obuli Sai Naren. Multi Cancer Dataset Cervical Cancer Kaggle (2022). 10.34740/KAGGLE/DSV/3415848.

[31] Obuli Sai Naren. Multi Cancer Dataset Lymphoma Kaggle (2022). 10.34740/KAGGLE/DSV/3415848.

[32] Plissiti, M. E. et al. Sipakmed: a new dataset for feature and image based classification of normal and pathological cervical cells in pap smear images. In *2018 25th IEEE international conference on image processing (ICIP)* 3144–3148 (IEEE, 2018). 10.1109/ICIP.2018.8451588.

[33] Orlov, N. V. et al. Automatic classification of lymphoma images with transform-based global features. *IEEE Trans. Inf Technol. Biomed.* **14** (4), 1003–1013. 10.1109/TITB.2010.2050695 (2010). 20659835 10.1109/TITB.2010.2050695 PMC2911652

[34] Simonyan, K. & Zisserman, A. Very deep convolutional networks for large-scale image recognition. *arXiv preprint arXiv:1409 1556*. 10.48550/arXiv.1409.1556 (2014).

[35] Mirjalili, S. & Lewis, A. The whale optimization algorithm. *Adv. Eng. Softw.* **95**, 51–67. 10.1016/j.advengsoft.2016.01.008 (2016).

[36] Felix, H. G. Recent advances and real-world implementations of the Whale Optimization algorithm. *Qubahan Technol. J.* **4** (2), 1–16. 10.48161/qtj.v4n2a52 (2025).

[37] Medjahed, S. A., Saadi, T. A., Benyettou, A. & Ouali, M. Gray wolf optimizer for hyperspectral band selection. *Appl. Soft Comput.* **40**, 178–186. 10.1016/j.asoc.2015.09.045 (2016).

[38] Makhadmeh, S. N. et al. Recent advances in Grey Wolf Optimizer, its versions and applications. *IEEE Access.* **12**, 22991–23028. 10.1109/ACCESS.2023.3304889 (2023).

[39] Gad, A. G. Particle swarm optimization algorithm and its applications: a systematic review. *Arch. Comput. Methods Eng.* **29** (5), 2531–2561. 10.1007/s11831-021-09694-4 (2022).

[40] Aguerchi, K., Jabrane, Y., Habba, M., Hassani, E. & A. H A CNN hyperparameters optimization based on particle swarm optimization for mammography breast cancer classification. *J. Imaging*. **10** (2), 30. 10.3390/jimaging10020030 (2024). 38392079 10.3390/jimaging10020030 PMC10889268

[41] Alibrahim, H. & Ludwig, S. A. Hyperparameter optimization: comparing genetic algorithm against grid search and bayesian optimization. In *2021 IEEE Congress on Evolutionary Computation (CEC)* 1551–1559 (2021). 10.1109/CEC45853.2021.9504761.

[42] Eligüzel, N., Cetinkaya, C. & Dereli, T. A novel approach for text categorization by applying hybrid genetic bat algorithm through feature extraction and feature selection methods. *Expert Syst. Appl.* **202**, 117433. 10.1016/j.eswa.2022.117433 (2022).

[43] Dorigo, M., Maniezzo, V. & Colorni, A. Ant system: optimization by a colony of cooperating agents. *IEEE Trans. Syst. Man. Cybern. Part. b (cybernetics)* **26** (1), 29–41. 10.1109/3477.484436 (1996). 10.1109/3477.484436 18263004

[44] Eido, W. M. & Ibrahim, I. M. Ant Colony Optimization (ACO) for traveling salesman problem: a review. *Asian J. Res. Comput. Sci.* **18** (2), 20–45. 10.9734/ajrcos/2025/v18i2559 (2025).

[45] Emara, A. H. M., Atteia, G. & Alkhateeb, J. H. Fine tuning hyperparameters of deep learning models using metaheuristic accelerated particle swarm optimization algorithm. *IEEE Access.*10.1109/ACCESS.2025.3591403 (2025).

[46] Mustafa, W. A. & Alquran, H. Editorial for the special issue advances in medical image processing, segmentation, and classification. *Diagnostics* **15** (9), 1114. 10.3390/diagnostics15091114 (2025). 40361932 10.3390/diagnostics15091114 PMC12071901

[47] Salmanpour, M. R. et al. Machine learning evaluation metric discrepancies across programming languages and their components in medical imaging domains: need for standardization. *IEEE Access.*10.1109/ACCESS.2025.3549702 (2025).

[48] Kaur, H., Sharma, R. & Kaur, J. Comparison of deep transfer learning models for classification of cervical cancer from pap smear images. *Sci. Rep.* **15** (1), 3945. 10.1038/s41598-024-74531-0 (2025). 39890842 10.1038/s41598-024-74531-0 PMC11785805

[49] Jadhav, V. S., Yakkundimath, R., Saunshi, G. & Konnurmath, G. Deep learning based classification of cervical cancer stages using transfer learning models. *Indian J. Med. Pediatr. Oncol.* **46** (05), 480–488. 10.1055/s-0045-1809907 (2025).

[50] Mondal, J., Chatterjee, R., Kumar Gourisaria, M., Sahni, M. & León-Castro, E. Cervical cancer classification using a novel hybrid approach. *Front. Oncol.* **15**, 1703772. 10.3389/fonc.2025.1703772 (2025). 41426318 10.3389/fonc.2025.1703772 PMC12711500

[51] Ogundokun, R. O., Owolawi, P. A., Tu, C. & van Wyk, E. Autoencoder-assisted stacked ensemble learning for lymphoma subtype classification: a hybrid deep learning and machine learning approach. *Tomography* **11** (8), 91. 10.3390/tomography11080091 (2025). 40863882 10.3390/tomography11080091 PMC12389832

[52] Elaraby, A., Nechaevskiy, A. & Saad, A. Advancing lymphoma diagnosis in histopathology image classification using multi deep learning models. *Iraqi J. Comput. Sci. Math.* **6** (2), 13. 10.52866/2788-7421.1251 (2025).

[53] Mahapatra, A. K., Panda, N., Mahapatra, M., Jena, T. & Mohanty, A. K. A fast-flying particle swarm optimization for resolving constrained optimization and feature selection problems. *Cluster Comput.* **28** (2), 91. 10.1007/s10586-024-04750-7 (2025).

[54] Mahapatra, A. K., Panda, N. & Pattanayak, B. K. Quantized orthogonal experimentation SSA (QOX-SSA): a hybrid technique for feature selection (FS) and neural network training. *Arab. J. Sci. Eng.* **50** (2), 1025–1056. 10.1007/s13369-024-09113-3 (2025).

[55] Mahapatra, A. K., Panda, N. & Pattanayak, B. K. Adaptive dimensional search-based orthogonal experimentation SSA (ADOX-SSA) for training RBF neural network and optimal feature selection. *J. Supercomput.* **81** (1), 212. 10.1007/s11227-024-06507-w (2025).

[56] Agrawal, U. K., Panda, N., Tejani, G. G. & Mousavirad, S. J. Improved salp swarm algorithm-driven deep CNN for brain tumor analysis. *Sci. Rep.* **15** (1), 24645. 10.1038/s41598-025-09326-y (2025). 40634405 10.1038/s41598-025-09326-y PMC12241476

[57] Agrawal, U. K. & Panda, N. Quantum-inspired adaptive mutation operator enabled PSO (QAMO-PSO) for parallel optimization and tailoring parameters of Kolmogorov–Arnold network. *J. Supercomput.* **81** (14), 1310. 10.1007/s11227-025-07810-w (2025).

